# Early lymph node T follicular helper cell signalling hub drives influenza vaccine response in an ancestrally diverse cohort

**DOI:** 10.1016/j.ebiom.2025.106036

**Published:** 2025-11-29

**Authors:** Jacqueline H.Y. Siu, Sofia Coelho, Aime Palomeras, Sandra Belij-Rammerstorfer, Ninisha Barman, Chloe H. Lee, Tamara Ströbel, Christopher J. Thorpe, Charandeep Kaur, Tom Cole, Nico Remmert, Jamie Fowler, Sam Pledger, Kyla B. Dooley, Terrence Chan, Katja Höschler, Maria Zambon, Daniel Opoka, Tamas Szommer, Seung J. Kim, Vinod Kumar, Samantha Vanderslott, Pontiano Kaleebu, Anita Milicic, Donald B. Palmer, Teresa Lambe, Brian D. Marsden, Hashem Koohy, Mark Coles, Calliope A. Dendrou, Katrina M. Pollock

**Affiliations:** a Kennedy Institute of Rheumatology, Nuffield Department of Orthopaedics, Rheumatology and Musculoskeletal Sciences, University of Oxford, Oxford, UK; b Centre for Medicines Discovery and Centre for Human Genetics, Nuffield Department of Medicine, University of Oxford, Oxford, UK; c NIHR Imperial Clinical Research Facility, Hammersmith Hospital, London, UK; d Oxford Vaccine Group, Department of Paediatrics, University of Oxford, Oxford, UK; e MRC Translational Immune Discovery Unit, Weatherall Institute of Molecular Medicine, Radcliffe Department of Medicine, University of Oxford, Oxford, UK; f European Molecular Biology Laboratory, European Bioinformatics Institute (EMBL-EBI), Wellcome Genome Campus, Hinxton, UK; g Pandemic Sciences Institute, Nuffield Department of Medicine, University of Oxford, Oxford, UK; h Respiratory Virus Unit, UK Health Security Agency, UK; i Medical Research Council/Uganda Virus Research Institute, and London School of Hygiene and Tropical Medicine, Uganda Research Unit, Entebbe, Uganda; j The Jenner Institute, Nuffield Department of Medicine, University of Oxford, Oxford, UK; k Department of Comparative Biomedical Sciences, Royal Veterinary College, London, UK; l NIHR Oxford Biomedical Research Centre, Oxford, UK

**Keywords:** Human lymph node fine needle aspirates, T follicular helper cells, Single-cell multi-omics, Ancestral diversity, Influenza, Vaccination

## Abstract

**Background:**

Early *in vivo* dynamics of human immune-cell activation across regionally activated lymphoid tissue sites upon immunisation are poorly characterised in ancestrally-diverse individuals with consequences for pandemic preparedness.

**Methods:**

In this experimental medicine study, draining and non-draining lymph nodes (dLNs and ndLNs) were studied by ultrasound (US)-guided fine-needle aspiration (FNA) in 13 adults aged 18–55 years with African and Asian ancestry, before and after receiving adjuvanted seasonal influenza vaccine (aQIV). A multi-modal investigation of ultrasound data, genotyping, systems serology, and single-cell multi-omics was undertaken.

**Findings:**

HLA subtypes reflected self-declared ethnicity and included understudied alleles. Draining but not ndLNs rapidly increased in size post-vaccination, by day 3, with distinct cellular dynamics culminating in a cross-protective serological response. Dissecting LN cellular diversity into 42 lymphoid and non-lymphoid cell states, early post-vaccination cell abundance changes were observed across all LNs, but dLNs were characterised by CD4^+^ T follicular helper (CD4^+^ Tfh) cell expansion. Gene expression analysis revealed a dLN post-vaccination hub defined by CD4^+^ Tfh signalling, cross-compartmental activation, translation, and enhanced antigen-presentation capacity.

**Interpretation:**

Early CD4^+^ Tfh coordination in draining lymphoid tissue underpins robust responses to adjuvanted influenza vaccine that transcend ancestral inter-individual variation in young adults, with implications for vaccine design in ancestrally-diverse populations.

**Funding:**

The study was funded by the 10.13039/100000923Silicon Valley Community Foundation with a Chan Zuckerberg Initiative donation. The funder had no role in the study design, data analysis or decision to publish. The funder provided infrastructure support for the posting of the dataset with CELLxGENE.

**Research in context**

**Evidence before this study**

Descriptions of B-cell evolution and T helper cell responses to non-adjuvanted influenza and COVID-19 vaccination weeks and even months after immunisation in the draining lymph node (dLN) but not the non-draining lymph node (ndLN) have been reported without specific focus on ancestral diversity and without prior investigation of adjuvanted vaccines. Pre-developed pandemic influenza vaccines include adjuvanted vaccines such as those containing the squalene adjuvant MF59C.1, but seasonal influenza vaccines for young adults are not adjuvanted. Our understanding of the early cellular kinetics of LN activation and its coordination across LNs located at draining and non-draining anatomical sites is thus incomplete and has not been investigated in ancestral diversity or in the setting of an adjuvanted influenza vaccine.

**Added value of this study**

This study offers a publicly available reference dataset reporting the early gross anatomical, cellular, and molecular dynamics of the draining and non-draining axillary lymph nodes of 13 individuals with African or Asian ancestry aged 18–55 years, after intramuscular immunisation with an adjuvanted quadrivalent seasonal influenza vaccine, aQIV (ISRCTN13657999). Comprehensive ancestral data including participant ancestral history across three generations, genotyping, and human leucocyte antigen (HLA) tissue typing were collected, resulting in 13 rarely described *HLA-B* alleles. The comparison of the dLN and ndLN cellular and molecular profiles revealed a rapid (days 3–7) increase in CD4^+^ T follicular helper (Tfh) cells, swelling of the dLN but not the ndLN, and a dLN multicellular gene expression hub that was dominated by CD4^+^ Tfh cell signalling and enhanced antigen presentation. Subsequent influenza-specific antibody induction was robust, transcending the observed HLA diversity. Encouragingly, whilst the aQIV included only seasonal influenza haemagglutinins, cross-reactive antibody responses against avian influenza were also induced. Anatomical, temporal and genomic coordination of cellular and molecular events dominated by CD4^+^ Tfh in the draining, but not the non-draining, lymph node is characteristic of an optimal immune response to adjuvanted seasonal influenza vaccine in young adults with African or Asian ancestry.

**Implications of all the available evidence**

This study provides a detailed description of the early immune dynamics in the critical responding tissue, and differentiation from the response in distal secondary lymphoid tissue after vaccination. Temporal, anatomical and cellular coordination was focused on the CD4^+^ Tfh response in the first week, in this ancestrally diverse cohort of young African and Asian adults, making these cells an important target for vaccine design. By making use of aQIV, which is not currently licenced for adults under the age of 65 years, the results provide evidence that an adjuvanted pandemic influenza vaccine could rapidly induce cross-protective antibody even in the setting of diversity with rarely described HLA-subtypes in the vaccinated population. Discovery of vaccines and therapeutics such as monoclonal antibodies is increasingly dependent on data that describe the human *in vivo* immune response. Development of new foundational tools, such as machine learning, cannot rely on existing reference datasets with their inherent ancestral and homoeostatic biases. The publicly available data arising from this study move towards addressing this need by providing a valuable resource for basic and translational immunity research in ancestral diversity, with important implications for pandemic preparedness.

## Introduction

The cellular and molecular mechanisms underpinning vaccine-induced health protection from respiratory viruses with pandemic potential, such as influenza, in African and Asian populations are rarely investigated, despite different vulnerability to severe disease.^1^ As a result, publicly available reference datasets such as the 1000 Genomes Project and the Human Cell Atlas are not ethnically representative, undermining efforts to develop improved seasonal, pandemic or universal influenza vaccines and therapeutics.^2^ Variation in inflammatory and immune responses in individuals with different ethnic backgrounds has been documented, with likely implications for the susceptibility and severity of response to pathogens.^3^^,^^4^ With respect to globally prevalent seasonal infections, such as influenza, respiratory mortality associated with the virus is highest in sub-Saharan Africa (2.8–16.5 per 100,000 individuals) and South-East Asia (3.5–9.2 per 100,000 individuals) compared with Europe (3.1–8.0 per 100,000 individuals).^1^ This may be attributed to geographic, socioeconomic and/or genetic factors, but notably, the risk of influenza infection and of severe outcomes following infection is also higher in individuals of Black, South Asian or mixed ethnicity residing in England and in the United States of America (USA).^5^, ^6^, ^7^ Similarly, non-white ethnicities were disproportionately represented amongst working-age adults with severe sequelae from the COVID-19 pandemic in the United Kingdom (UK) and USA.^8^^,^^9^ Research addressing the immunological factors that underpin these differences—such as population-dependent human leucocyte antigen (HLA) diversity, variation in early events in immune cell activation in the critical responding secondary lymphoid tissue, and the orchestration of responses across the lymphatic network—is required to inform the design of vaccines and immunotherapeutics to help overcome health vulnerability and to boost pandemic preparedness.

Lymph node (LN) fine-needle aspiration (FNA) is emerging as a tractable methodology to address unexplored aspects of human lymphatic biology *in vivo*, which is critical to vaccine-induced immunity, during longitudinal studies.^10^ Within LNs, germinal centre (GC) interactions between B cells and CD4^+^ T follicular helper (CD4^+^ Tfh) cells drive the high-affinity antibody responses typically required for vaccine-dependent, long-term protection against pathogens. Studies in animal models suggest that early LN immune responses dictate vaccine priming efficacy, but in humans the early *in vivo* dynamics of immune-cell activation in secondary lymphoid tissues upon immunisation remain poorly characterised.^11^ Instead, human research in vaccination relies on peripheral blood samples, and whilst the frequency and activation of circulating CD4^+^ Tfh cells and plasmablasts increase in young adults within the first week post-immunisation with recall antigens, the blood does not contain GCs.^12^^,^^13^ Recent studies employing ultrasound (US)-guided FNA to sample draining LNs (dLNs) have utilised FNA-derived dLN cell profiling to demonstrate the evolution of B and CD4^+^ Tfh cell responses to non-adjuvanted influenza and COVID-19 mRNA vaccination weeks and even months after immunisation.^14^, ^15^, ^16^, ^17^, ^18^

Several questions remain unaddressed, however, including the early cellular kinetics of LN activation, and their coordination across LNs located at different anatomical sites. It is not understood how this process is influenced by an adjuvant, such as MF59C.1, used in adjuvanted quadrivalent influenza vaccine (aQIV). In the event of an avian influenza pandemic, an H5N1 influenza vaccine including the MF59C.1 adjuvant could be deployed, but how this formulation induces immunity in young adults is not well studied, as it is not used in this population for seasonal immunisation.

To address the paucity of LN data from individuals of non-European ancestry, and from multiple LN sites sampled simultaneously, we have profiled LNs before and after vaccination with the 2022–2023 seasonal quadrivalent influenza vaccine adjuvanted with MF59C.1 in a cohort of young adults of African or Asian ancestry recruited from the West London area in the UK as part of the ‘Lymph nodE single-cell Genomics AnCestrY’ (LEGACY) Network. FNA was performed to obtain cells from both the axillary draining and non-draining LNs (dLNs and ndLNs, respectively) for analysis by single-cell multi-omics. The data arising provide an important open resource with ethnic representation of LN cells at the genetic, transcriptomic and protein level and show the early anatomical, temporal and cellular dynamics of human axillary LNs after intramuscular immunisation in ancestrally diverse young adults.

## Methods

### Clinical study

#### Ethics

The full version of the approved clinical study protocol, version 2.1, 09 Aug 2022, is available.^19^ The study is registered under ISRCTN13657999. The study was approved by the London—Central Research Ethics Committee (REC ref: 22/LO/0343). The sponsor was Imperial College London. This was a single-site interventional non-randomised open label experimental medicine clinical research study in 30 volunteers in total over two influenza seasons (2022–2023 and 2023–2024). Volunteers provided written informed consent to participate in the study. The study was run at the National Institute for Health and Care Research (NIHR) Imperial Clinical Research Facility, West London, UK. We report here data from the first season cohort, who completed all study visits in 2022–2023 (*n* = 13).

#### Eligibility and enrolment

Participants were eligible to volunteer for the study if they: (i) were healthy adults aged ≥18 years and ≤55 years on the day of screening, (ii) were willing and able to provide written informed consent, (iii) identified as having African or Asian ancestry, (iv) were usually resident in the UK for ≥5 years prior to screening, (v) were not pregnant on the day of screening and were willing to use a highly effective form of contraception until 12 weeks after the study immunisation (if a person of childbearing potential), (vi) were willing to avoid all other vaccines within 4 weeks either side of the study injection and fine needle aspiration, (vii) were willing and able to comply with the visit schedule and provide samples, and (viii) were willing to grant authorised persons access to their trial related medical record and GP records either directly or indirectly. Participants were given written information about the study and the opportunity to ask questions. Those wishing to take part gave written informed consent and underwent screening procedures including a general health check, medical history assessment and blood tests to confirm eligibility. Participants’ sex was self-reported. Participants who were pregnant or lactating, had a significant medical history, a body mass index of ≥30, a history of allergy to drugs/vaccines, anaphylaxis or angioedema, had recently (within 18 weeks) taken immunosuppressive agents, or were taking part in another trial were not eligible. Participants underwent screening tests for blood borne viruses and were ineligible if they had detectable antibody to HIV, hepatitis C or hepatitis B. All participants completed the study save one additional participant who was enrolled but subsequently withdrew.

#### Safety

There were no safety objectives of this experimental medicine study. To ensure the safety and well-being of participants safety assessments were conducted as follows. During the study, participants were asked about concomitant medications at every visit and about COVID-19 symptoms at every in person visit. People of childbearing potential had a urinary pregnancy test at screening and on the day of immunisation and were asked to use an effective form of contraception during the study if sexually active. A symptom directed physical examination was performed where necessary. Adverse events were recorded in the first 5 days following LN tissue sampling and in the 28 days following influenza vaccination for the study. Serious adverse events were collected throughout the study. Side effects of the vaccination that were suspected adverse reactions were reported to the MHRA through the yellow card scheme by the investigator. Participants provided written, informed consent and were monitored for safety throughout; there were no serious adverse events and no unexpected adverse reactions to vaccination. Adverse events occurring post FNA were all mild (grade 1) self-limiting, and as expected; axillary bruising, pain or tenderness at the FNA site. Axillary swelling and axillary redness were reported once.

#### Study immunisation

The study immunisation, adjuvanted quadrivalent influenza vaccine (aQIV) (CSL Seqirus), was administered as an intramuscular injection into the non-dominant arm (as requested by study participants) in the deltoid muscle. One 0.5 mL dose of aQIV contained 15 μg of haemagglutinin from two A and two B strains of influenza propagated in hens’ eggs and adjuvanted with MF59C.1. This contained per 0.5 mL dose, squalene (9.75 mg), polysorbate 80 (1.175 mg), sorbitan trioleate (1.175 mg), sodium citrate (0.66 mg) and citric acid (0.04 mg). The 2022/2023 UK recommendation for influenza vaccine study composition was: A/Victoria/2570/2019 (H1N1)pdm09-like virus, A/Darwin/9/2021 (H3N2)-like virus, B/Austria/1359417/2021-like virus, and B/Phuket/3073/2013-like virus. The aQIV clinical study vaccine contained: A/Victoria/2570/2019 IVR-215, A/Darwin/6/2021 IVR-227, B/Austria/1359417/2021 BVR-26, and B/Phuket/3073/2013 BVR-1B.

#### Ultrasound-guided fine-needle aspiration sampling procedure

The US-guided FNA was taken by a qualified medical practitioner according to our established standard operating procedure. Participants were ineligible to undergo the procedure if they were taking blood thinning medication prior to aspiration, if there were signs of local infection, or if there was pain or swelling at the site of sampling. The FNA was conducted using standard aseptic technique under US guidance using a Toshiba Aplio i700 US machine. Following infiltration of local anaesthetic, a sterile 21G needle and syringe were inserted into the axillary lymph node under US guidance using 3–5 passes. Aspirated cells were placed directly into sterile-filtered RPMI 1640 solution with HEPES, heat-inactivated foetal bovine serum, penicillin-streptomycin and l-glutamine (R10) for transport to the laboratory in a sealed air-sea container with cooled gel packaging.

### Sample processing

Samples were processed immediately on receipt, as previously described for viable cryopreservation.^19^^,^^20^ LN FNA samples were processed as previously published.^20^ In brief, LN FNAs were collected in R10 media and transported at 4 °C to the laboratories. R10 media contains RPMI 1640 with 25 mM HEPES (Cat No: R5886, Sigma), 10% heat-inactivated foetal bovine serum (HI-FBS) (Cat No: F4135, Sigma), 1% Pen/Strep (Penicillin-Streptomycin 10,000 Units/mL (Cat No: 15140-122, Gibco), and 2 mM of l-Glutamine (Cat No: 25030-024, Gibco). Samples were washed in RPMI with 5% heat-inactivated and filtered human AB serum (Sigma-Aldrich, Cat No: H4522), incubated in ACK lysis buffer (Gibco, Cat: A10492-01) at room temperature for 5–10 min, and then washed twice with cell wash buffer (PBS + 2% human AB serum + 2 mM EDTA). Cells were then cryopreserved in CryoStor CS10 (Stemcell Technologies, Cat No: 07930) in aliquots of 500,000 cells in 100 μL in the vapour phase of liquid nitrogen (−160 °C).

### Ultrasound analysis

Images of sampled LNs, typically six per axillae, were taken at the FNA visits before and after immunisation. Analysis of images was undertaken by two medical practitioners confirming the clearest image of the sampled node, which was then measured in two axes in the cross-sectional plane. The long-axis diameter measurement was utilised as a metric of lymph node size.

### Genotyping, HLA typing and HLA peptide-binding estimation

DNA was isolated from whole blood using the QIAamp DNA Blood Midi Kit according to manufacturer's instructions. Genotyping was performed using the Infinium Global Screening Array-24 v3.0 (Illumina) by the Genome Centre, Queen Mary University of London according to manufacturer's recommendations. This dataset contained 730,000 polymorphic variants. HLA typing, specifically HLA class I *HLA-A/B/C* (exons 2 and 3) and class II *DRB1/DQB1* (exon 2 only) up to four digits, was performed by the sequencing/typing facility at the MRC Weatherall Institute of Molecular Medicine, University of Oxford. LEGACY cohort HLA allele frequency was compared to the 1000 Genomes Project HLA data (RRID: SCR_008801)^21^ derived from five ancestral super populations (African, *n* = 712; Admixed American, *n* = 373; European, *n* = 529; South Asian, *n* = 543; and East Asian, *n* = 536 individuals). The count of peptides known to bind to different *HLA-A/B/C* and *HLA-DRB1* alleles was obtained and plotted from estimates provided by the Immune Epitope Database (RRID: SCR_006604), accessed on the 11th of August 2024.^22^

### Influenza strain HA-specific IgG standardised ELISA

Standardised ELISA was used to quantify circulating influenza strain HA-specific IgG responses. Briefly, ELISA plates were coated overnight with either 2 μg/mL of A/Victoria/2570/2019 HA, A/Darwin/9/2021 HA, B/Austria/1359417/2021 HA, B/Phuket/3073/2013 HA (Native Antigen: REC32023-500, REC32002-500, REC32004-500 and REC32022-500, respectively). After blocking with Blocker Casein in PBS (Thermo Fisher Scientific), samples (minimum 1:100 dilution) were incubated for 2 h at room temperature. Standard curve and internal controls were created from reference serum using a pool of high-titre donor serum. An alkaline phosphatase-conjugated secondary antibody (Merck A3187, RRID: AB_258054) was then added and incubated for 1 h at room temperature. Plates were developed using PNPP alkaline phosphatase substrate (Thermo Fisher Scientific) and read at 405 nm when the internal control reached an optical density of 1. Friedman tests were used to assess statistical significance in antibody levels by time point, with a significance threshold of *P*-value <0.05.

### Multiplexed immunoassay

Antigen-specific IgG subclasses, IgM and IgA binding profiles were generated using a custom magnetic multiplexed immunoassay on the Luminex platform. Antigens (A/Victoria/2570/2019 HA, A/Darwin/9/2021 HA, B/Austria/1359417/2021 HA, B/Phuket/3073/2013 HA from Native Antigen: REC32023-500, REC32002-500, REC32004-500 and REC32022-500, respectively) were coupled to Bio-Plex Pro Magnetic COOH beads (Bio-Rad) using the Bio-Rad Bio-Plex Amine Coupling Kit. Using a black, clear-bottom 96-well plate (Greiner), 1000 beads per bead region and samples (minimum 1:100 dilution) were added per well. The plate was covered and incubated at 37 °C on a shaker for 1 h and was then washed with PBS containing 0.05% Tween20 (PBST). Antigen-specific antibodies were detected using phycoerythrin (PE)-conjugated mouse anti-human IgG1-4, IgA1-2 and IgM (Southern Biotech; RRIDs: AB_2796628, AB_2796639, AB_2796701, AB_2796693, AB_2796656, AB_2796664, AB_2796577). After incubation at room temperature for 30 min on a shaker, the plate was washed before the beads were resuspended in 50 μL of sheath fluid. The plate was then incubated at room temperature for 10 min on a shaker before being read by the MAGPIX® System. The binding of the PE-detectors was measured to calculate the median fluorescence intensity. Friedman tests were used to assess statistical significance in antibody levels by time point, with a significance threshold of *P*-value <0.05.

### Haemagglutination Inhibition

Serum samples were analysed by Haemagglutination Inhibition (HAI) with the viruses used in the 2022/23 influenza vaccine: A/Victoria/2570/2019 (H1N1) pdm09-like strain, A/Darwin/9/2021 (H3N2)-like strain, B/Austria/1359417/2021-like strain, B/Phuket/3073/2013-like strain. Serum was pre-treated with neuraminidase followed by heat inactivation for 1 h at 56 °C. Samples were titrated in an 8-step two-fold dilution series, incubated with the HA antigen suspension for 1 h at room temperature, and 25 μL of the 0.5% RBC suspension (turkey blood for the H1N1 and B strains; guinea pig blood for the H3N2 strain) added. The reaction was left for up to 1 h before reading.

Titre duplicates for each serum-virus combination achieved less than two-fold variance or were repeated. The standard dilution series covered titres 10–1280 with samples which did not reach an endpoint in this initial titration undergoing endpoint titration; titres which were considered to indicate sero-protection were HAI titres ≥40. Friedman tests were used to assess statistical significance by time point, with a significance threshold of *P*-value <0.05.

### Serology and metadata correlation analysis

Pearson correlation of serological log_2_ fold-change post-vaccination response compared to baseline with participant metadata including age, BMI, draining and non-draining LN diameter before and after vaccination was performed (615 correlations total) and FDR was used to correct for multiple comparisons.

### Single-cell experiments

LN FNA samples, pre-stained with TotalSeq-C Human Universal Cocktail V1.0 (BioLegend; RRID: AB_2876728) when cell numbers allowed (as per the manufacturers' guidance), were loaded with a maximum of 20,000 cells onto 10x Genomics Chromium X controller with standard kits. Libraries (5′ gene expression, V(D)J, and ADT) were prepared using manufacturers’ guidelines and sequenced using NovaSeq 6000 PE150 for LN FNA (RRID: SCR_016387).

### Computational analyses

#### Genotyping quality control and admixture

The input array data consisted of 13 samples and 730,000 variants prior to filtering and QC. Filtering and QC steps included: (1) retaining only autosomal variants, (2) removing loci where 99.9% of genotypes are missing, (3) removing SNPs with minor allele frequency of 1%, and (4) excluding variants with one or more multi-character allele code. The final post-QC array data consisted of 330,000 variants. The reference dataset of merged genotypes combined samples from 1000 Genomes and HGDP.^2^^,^^23^ Intersecting variants between our data and the reference data for downstream analysis was determined by the following: (1) exclude non-unique SNPs, (2) exclude A-T or G-C SNPs, and (3) aligned position mismatches and allele flips. The program ADMIXTURE (v.1.3.0; RRID: SCR_001263) was used to estimate per-individual ancestry populations in a panel of 3433 reference individuals representing African, European, East Asian, and American ancestries.^24^ The optimum number of ancestry populations (K) was chosen based on five-fold cross-validation for each K in the set of 5–30. Model of K = 18 had the lowest cross-validation error. The population allele frequencies estimated from the analysis of reference samples were fixed as parameters so that the LEGACY samples could be projected into the admixture model to obtain ancestry proportion estimates.

#### Bayesian modelling

Bayesian hierarchical linear models were used to examine two relationships: firstly, the effect of pre-vaccination IgG titres on post-vaccination responses, and secondly, the contribution of IgG subtypes to total IgG titres across strains. In the first model, post-vaccination IgG titres (natural-log-transformed) were regressed on pre-vaccination titres measured at the pre-vaccination and day 0 time points. In the second model, total IgG titres (natural-log-transformed) were regressed on IgG subtype levels (IgG1–4, natural-log-transformed), with study time point, strain, and their interactions included as predictors. Both models incorporated individual-level random intercepts and were adjusted for age, sex, body mass index (BMI), and self-reported ethnicity as fixed effects. Weakly informative priors were specified for all fixed and random effects.^25^^,^^26^ Posterior estimates were summarised using 95% credible intervals calculated from highest posterior density intervals. Models were implemented in R using the brms package^27^^,^^28^ with cmdstanr^29^ and cmdstan version 2.34.1 serving as interfaces to the Stan probabilistic programming language.^30^

#### Principal component analysis (PCA)

PCA was performed using donor characteristics, sampling cell counts, and measured LN diameter. Prior to performing PCA, missing data were imputed using imputePCA (missMDA package) with number of components used equal to half the number of variables used in the PCA. PCA results were visualised using factoextra package (RRID: SCR_016692).

#### Single-cell pre-processing, quality control to clustering and annotation

From the Chromium single-cell RNA sequencing outputs, Cell Ranger v7.0 was used for transcript alignment to GRCh38 version 2020-A and generation of feature-barcode matrices for downstream analysis. Panpipes were used for quality control, batch correction, dimension reduction, and clustering.^31^ High-quality single cells were identified by removing doublets determined using Scrublet^32^ (RRID: SCR_018098), cells expressing fewer than 300 genes, cells with mitochondrial gene count percentage greater than 25%, cells with haemoglobin gene count percentage greater than 40%. Genes detected in fewer than 3 cells were also removed.

UMI counts were normalised using the shifted logarithm technique found in scanpy's pp.normalized_total function (RRID: SCR_018139). Top 3000 highly variable genes, excluding *IGHV* and *TCR* genes, were selected using VST from Seurat v3 (RRID: SCR_016341) for downstream dimension reduction. Harmony (RRID: SCR_018809) was used for batch correction between experimental batches with 30 PCs. The results were then visualised in a lower dimension using UMAP. Leiden clustering helped identify three broad cell populations: T/NK/IL cells, B/plasma cells, and non-lymphocytes. These broad populations were then individually further clustered with re-calculated highly variable genes to generate finer annotations. Clusters with mixed gene signatures (e.g., co-expressing canonical T and B cell genes) were further clustered if possible but otherwise removed if deemed to have a high likelihood of being a doublet by the Scrublet score. The Wilcoxon rank-sum test was used to determine the top expressed genes in each cluster to aid with annotation.

#### Differential abundance analysis

MASC (mixed-effects modelling of associations of single-cells [RRID: SCR_025632])^33^ was used to identify differentially abundant cellular populations associated between conditions whilst accounting for participant's weight and the number of days post-vaccination that the second LN FNA sample was collected as fixed effects. Conditions compared include dLN pre- vs post-vaccination, ndLN pre- vs post-vaccination, and post-vaccination dLN vs ndLN.

#### SingleR reference mapping

SingleR^34^ was used to transfer annotations between our dataset and a comparable publicly available LN FNA dataset (10.5281/zenodo.6476022).^17^ To ensure comparable analysis, samples included were limited to analogous time points to our study (≤7 days post-vaccination) and the highest annotation resolution available. Both our dataset and the publicly available LN FNA dataset were used as both reference and test datasets to transfer annotations bi-directionally using the Wilcoxon differential expressed method at a single-cell resolution.

#### Single-cell TCR analysis

The TCR repertoire analysis used the Immcantation and scRepertoire (RRID: SCR_025691) frameworks. V, D, and J genes were assigned using IgBlast (RRID: SCR_002873). Non-productive sequences, cells with more than 3 alpha or beta chains, and those with gamma-delta constant regions were removed. Clones were identified using both VDJC gene and CDR3 nucleotide. Normalised Shannon entropy and expansion frequency (proportion of cells in a clone of two or more cells) were calculated using R package scRepertoire outputs.

To examine clonal overlaps of TCR repertoires between populations, we computed Morisita's index (MI) using the R package divo for all pairwise T-cell clusters. For shared clonal network analysis and visualisation, the R package igraph (RRID: SCR_021238) was used to construct weighted, undirected T-cell networks, with edge weights defined by Morisita's clone overlap index, node colour denoting clonality, and node size denoting number of clones. For clarity of cell networks, we filtered out edges with very low clonal overlap. We defined these filter thresholds based on the distribution of Morisita's index across all CD4^+^ and CD8^+^ T-cell populations, as these are likely to be spurious connections, considering all edges below the 95th percentile as low confidence. Raw, unfiltered counts of all overlaps were visualised as heatmaps. Networks were visualised using the R package ggraph (RRID: SCR_021239), with graphs laid out for visualisation using a force-directed Fruchterman-Reingold layout.

#### Identification of gene expression programmes (GEPs) using consensus non-negative matrix factorisation (cNMF)

cNMF was used to simultaneously capture gene functional programs and activation states alongside cell identities^35^ (RRID: SCR_025495). For each of the four broad cell types (B/plasma cells, CD4^+^ T cells, CD8^+^ T/NK cells, and non-lymphocytes), cNMF was run using 300 iterations and 3000 highly variable genes. The selection of k (number of components or gene expression programmes [GEPs]) was determined based on stability of the solution by the silhouette score, Frobenius reconstruction error, and biological relevance of the top weighted genes. Models of k = 17, 17, 15, and 10 were chosen for B/plasma cells, CD4^+^ T cells, CD8^+^ T/NK cells and non-lymphocytes, respectively. Outliers in consensus solutions were then filtered out based on histogram of components and their nearest neighbours using density thresholds of 0.15, 0.25, 0.25, and 0.21 for B/plasma cells, CD4^+^ T cells, CD8^+^ T/NK cells and non-lymphocytes, respectively.

To directly compare distribution functions of GEP score expression, Wasserstein distance or Earth mover's distance (EMD) was used to compute the difference between GEP score histograms between sample conditions (i.e., visit type, vaccination side) with a bin size of 0.2. A large EMD score indicates a greater difference between the two sample distributions. To estimate the significance of the EMD scores for each GEP score per broad cell type, a permutation-based method was used to calculate a q-value. Specifically, using EMDomics package, the overall EMD score for each GEP is calculated by averaging all of the pairwise EMD scores and then labels randomly permuted 1000 times and the EMD score for each permutation is also calculated. The null distribution is determined by the median of the permuted scores for each GEP, and the FDR is determined for a range of thresholds. The threshold that minimises FDR is defined as the q-value. GEPs with significant q-values (q < 0.05) indicated that those programmes have different distributions among the four sample conditions; thus, these GEPs were used in downstream hub analysis.

#### Identification of hubs and enrichment upon vaccination

Hubs with covarying GEPs were identified and compared between vaccination conditions. First, programme activity of each GEP per sample was summarised as a quantile mean (qmean) across five quantiles (0.25, 0.5, 0.75, 0.95, 0.99). A Pearson correlation co-efficient (R) was calculated for each GEP pairing across all samples. Fisher transformation was applied to the correlations, and the correlation mean was used as a test statistic. A null distribution was determined by permuting the sample identity 10,000 times whilst keeping the cell type constant, and R was tested against this null distribution. The correlation p-value was determined by counting how often the permuted R value differed from the true R value. The minimum count was scaled by two, designated the p-value statistic, and adjusted for multiple comparisons by Benjamini-Hochberg FDR of 10%. An adjusted R value was determined by calculating the difference of the mean true R values and the mean of permuted R values. Hierarchical clustering was used to cluster the GEPs. To determine if a GEP varied significantly before and after vaccination on the draining and non-draining sides, respectively, the qmean (as described above) of each GEP per donor per sample condition was compared using Wilcoxon rank-sum test and adjusted for multiple comparisons using Benjamini-Hochberg FDR of 10%.

#### MAST differential gene expression analysis

MAST^36^ was used to compare post-vaccination to pre-vaccination LN samples as well as dLN to ndLN samples across cell subtypes (B cells, CD4^+^ T cells, CD8^+^ T cells, innate-like immune cells, myeloid cells, and non-immune cells). Genes expressed in >10% for each cell state were used. Covariates included donor to account for multiple samples per participant. Other parameters include method is “glmer”, ebayes is FALSE. Gene set enrichment analysis was performed on the ranked fold-change results using fgsea^37^ comparing hallmark and curated canonical pathway gene sets (C2 CP). Gene set size was between 15 and 400 genes.

### Statistics

Statistical tests for each analysis were chosen and executed as described above. No formal sample size calculation was carried out. The study was open-label without blinding, and there was no randomisation. Inclusion and exclusion criteria were as described above. All statistics, data analysis, and visualisation was done in Python (RRID: SCR_008394) or R (RRID: SCR_000432) unless otherwise stated. Other single-cell analysis figures were produced using R packages including ggplot2 (RRID: SCR_014601), ggpubr (RRID: SCR_021139), ComplexHeatmap (RRID: SCR_017270)^38^; Python packages including muon (RRID: SCR_022804)^39^; and the Panpipes workflow.^31^ Significance across figures is indicated as follows: ∗*P* < 0.05; ∗∗*P* < 0.01; ∗∗∗*P* < 0.001; ∗∗∗∗*P* < 0.0001. Schematics were produced using BioRender.com (RRID: SCR_018361).

### Role of funders

The study was funded by the Silicon Valley Community Foundation from a donation by the Chan Zuckerberg Initiative, the funder had no role in the study design, data collection, data analyses, data interpretation, report writing or decision to publish. The funder provided data infrastructure support for the posting of the online dataset with CELLxGENE.

## Results

### Rarely studied HLA types amongst ancestrally diverse participants responding to aQIV

**Fig. 1:**
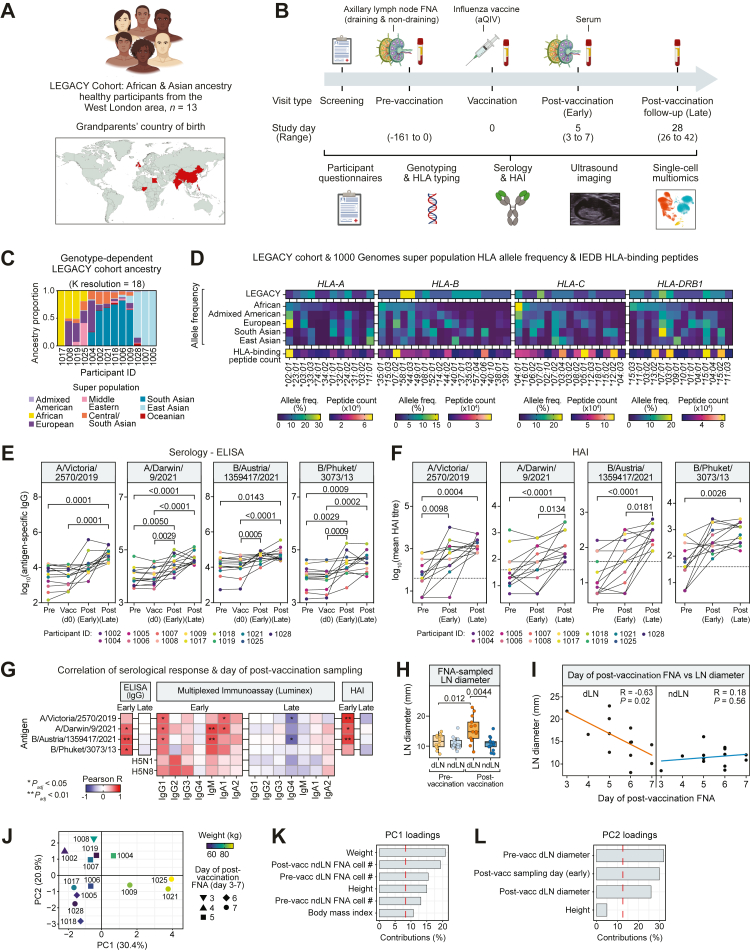
**LEGACY study design, cohort ancestry and serological and anatomical response.** (A) West London cohort of healthy participants who self-identified as having African and Asian ancestry (n = 13). Global map indicates the country of birth of the participants' grandparents. (B) Timeline of the study where multi-modal data is collected at various time points before and after influenza vaccination adjuvanted quadrivalent influenza vaccine (aQIV). (C) Cohort ancestry as determined by genotype-dependent admixture analysis. Stacked bars represent the admixture composition of each participant at K = 18. (D) Cohort HLA allele frequency relative to 1000 Genomes super populations and the count of peptides known to bind to these alleles based on the Immune Epitope Database (IEDB). (E) Detection of IgG antibodies against influenza strains used in the 2022/23 influenza vaccine by ELISA assay at various time points (n = 13, pre: pre-vaccination, vacc: day of vaccination, post (early): median five days post-vaccination, post (late): median 28 days post-vaccination). Differences in IgG levels by time point were assessed by the Friedman test; numbers above the brackets indicate significant *P*-values. (F) Haemagglutination inhibition (HAI) assay to identify functional antibody responses to the viruses used in the 2022/23 influenza vaccine. The dotted line represents the HAI seroconversion threshold (HAI ≥ 40). Differences by time point were assessed by the Friedman test (n = 13). (G) Correlation of serological response, as measured by ELISA, multiplexed immunoassay (Luminex), and HAI assay, and the day of post-vaccination sampling for the early and late sampling time points, corrected for multiple comparisons. Median and range of sampling days for the early and late time points are as shown in (B). R is the correlation coefficient; *P* is the *P*-value. (H) Diameter (mm) of FNA-sampled draining and non-draining lymph nodes (dLN and ndLN, respectively) before and after vaccination, as estimated from US images. Error bars represent the standard error. One-way ANOVA compared effect of sample location and time point with LN size. A Tukey HSD post-hoc test for multiple comparisons found that the mean of post-vaccination dLN diameter was significantly different compared to the other sample location and time points. (I) Linear correlation of dLN (left) and ndLN (right) estimated diameter with the number of days post-vaccination. R is the correlation coefficient; *P* is the *P*-value. (J–L) Principal component analysis (PCA) was conducted on pre- and post-vaccination (pre-vacc and post-vacc, respectively) LN cell counts, LN diameter, and donor characteristics such as weight (kg), height (cm), and BMI. (J) PCA coloured by donor weight (kg). Symbols indicate the day of post-vaccination FNA for each participant; numbers next to symbols denote the participant ID. Principal component (PC)1 and PC2 percentages indicate the variation captured by each component. (K, L) PC1 and PC2 loadings, showing parameters and donor characteristics with at least a 5% contribution to each PC. Dashed red threshold line represents the theoretical value if all variable contributions were equal.

Adult volunteers with self-declared African or Asian ancestry (n = 13) were enrolled and completed visits during the northern hemisphere winter flu season of 2022/23 (Fig. 1A). To collect steady state LN cells, US-guided FNA of axillary LN in both the left and right axillae was performed with paired collection of peripheral blood, prior to or at the start of the 2022/23 winter season. Participants received aQIV by intramuscular injection into the deltoid muscle of the arm. Arm selection for injection (left or right) was by participant choice and was recorded by the study team. Five (±2) days later (‘early’ post-vaccination time point) US-guided FNA of dLNs and non-draining LNs (ndLNs) was repeated, with a final collection of serum at a median of 28 days (range 26–42 days) after immunisation (‘late’ post-vaccination time point) (Fig. 1B).

Participants were 5 men and 8 women, with a median age of 34 years (confidence interval ±4.8 years). Participants reported a wide range of self-declared ethnicity including Middle Eastern, Asian, African, and mixed ethnicities (Fig. 1A; Table 1). The country of birth was most frequently United Kingdom/England, with others reported as France, China and India. All participants had been resident in the UK for at least 5 years before enrolling into the study. The ancestral diversity of the participants was evident through genome-wide genotyping and admixture analyses, demonstrating representation from across eight different ancestral super populations (Fig. 1C).

**Table 1:** Demographics and study details of LEGACY study participants.

Subject ID	Age (yr)	Sex	Day of post-vaccination FNA	No. of days between FNAs	Vaccine arm	Weight (kg)	Height (m)	BMI	Country of birth	HANCESTRO ontology
LEG1002	24	F	4	29	L	56.2	1.7	19.45	India	Indian
LEG1004	50	M	5	27	L	79.7	1.87	22.79	UK	Indian/Irish
LEG1005	21	F	7	44	L	58.5	1.71	20.01	China	Chinese
LEG1006	34	F	5	29	R	62.7	1.64	23.31	France	Tamil
LEG1007	22	F	5	50	L	68	1.67	24.38	China	Chinese
LEG1008	27	F	3	24	R	74.4	1.72	25.15	UK	British/Afro-Caribbean
LEG1009	35	M	6	48	R	85.5	1.77	27.29	UK	Bangladeshi
LEG1017	34	F	6	29	L	70	1.59	27.69	UK	Yoruban
LEG1018	38	M	7	42	L	61	1.59	24.13	UK	Indian
LEG1019	41	F	5	33	L	52.1	1.64	19.37	UK	British/Yoruban
LEG1021	40	M	6	13	L	89	1.78	28.09	India	Indian
LEG1025	32	M	6	14	L	93.8	1.86	27.11	UK	Arab
LEG1028	19	F	6	13	L	53.4	1.6	20.86	UK	Filipino

Study cohort demographics include age, self-reported sex, weight, height, body mass index (BMI), country of birth (standardised), and self-declared ethnicity (standardised to HANCESTRO ontology). Study details including day of post-vaccination FNA, number of days between LN FNAs, and vaccination arm. F, female; L, left; M, male; n.d., no data; R, right.

Participants also showed human leucocyte antigen (HLA) genotype diversity, with several rarely studied HLA genotypes being present in the cohort, as compared to the 1000 Genomes Project super population cohorts.^2^^,^^21^ HLA class I and II alleles were highly polymorphic across the cohort; for example, for the *HLA-B* locus, 18 discrete alleles were identified, of which 13 (72%) were rarely described alleles (Fig. 1D; Table 2). Given this HLA allele heterogeneity, we investigated the number of peptides that have been determined to bind to the different HLA alleles observed, using the Immune Epitope Database.^22^ The number of HLA-binding peptides characterised varied widely, spanning four orders of magnitude, with the highest peptide counts typically being found for HLA alleles having a high frequency in Europeans (Fig. 1D). For example, the *HLA-A* alleles with the highest frequency in Europeans (*HLA-A∗02:01*, *∗03.01*, *∗01:01*, *∗24:02*, and ∗*11:01*) had a significantly higher peptide binding count (Mann-Whitney test *P* = 0.0025), compared to the other *HLA-A* alleles in our cohort.

**Table 2:** LEGACY cohort HLA genotypes and genotype-dependent ancestral composition.

Subject ID	HLA-A∗ allele 1	HLA-A∗ allele 2	HLA-B∗ allele 1	HLA-B∗ allele 2	HLA-C∗ allele 1	HLA-C∗ allele 2	HLA-DRB1∗ allele 1	HLA-DRB1∗ allele 2	HLA-DRB3/4/5∗ allele 1	HLA-DRB3/4/5∗ allele 2	HLA-DQB1∗ allele 1	HLA-DQB1∗ allele 2	AMR	OCE	CSA	AFR	MDE	EAS	EUR	SAS
LEG1002	24:02	31:01	37:01	40:01	03:04	06:02	04:04	10:01	DRB4	DRB4	03:02	05:01	0.018	0.009	0.220	0.009	1.00E-05	0.012	0.096	0.637
LEG1004	02:01	33:03	08:01	44:03	07:01	07:01	03:01	07:01	DRB3	DRB4	02	02	0.009	0.009	0.149	0.002	1.00E-05	0.019	0.565	0.246
LEG1005	24:02	33:03	39:01	58:01	03:02	03:04	13:02	15:01	DRB3	DRB5	06:02	06:09	0.009	1.00E-05	2.00E-05	4.00E-05	1.00E-05	0.991	2.00E-05	1.00E-05
LEG1006	11:01	24:02	35:01	44:03	04:01	04:01	07:01	15:02	DRB4	DRB5	02	06:01	0.008	0.004	0.061	0.008	0.034	0.047	0.020	0.817
LEG1007	11:01	33:03	40:01	58:01	03:02	07:02	03:01	04:04	DRB3	DRB4	02	03:02	0.014	1.00E-05	2.00E-05	4.00E-05	1.00E-05	0.986	2.00E-05	1.00E-05
LEG1008	03:01	74:01	07:02	15:03	02:10	07:02	01:01	11:01	DRB3	n.f.	03:01	05:01	0.004	0.005	0.044	0.504	1.00E-05	4.00E-05	0.443	1.00E-05
LEG1009	32:01	33:03	44:03	49:01	07:01	07:01	07:01	11:03	DRB3	DRB4	02	03:01	0.028	0.010	0.064	0.001	0.016	0.133	0.099	0.649
LEG1017	01:01/04N	34:02	58:01	58:01	07:01	16:01	15:03	15:03	DRB5	DRB5	06:02	06:02	3.00E-05	3.49E-04	2.00E-05	1.000	1.00E-05	4.00E-05	2.00E-05	1.00E-05
LEG1018	01:01/04N	23:01	40:06	44:03	04:01	15:02	07:01	11:01	DRB3	DRB4	02	03:01	0.028	0.030	0.108	0.007	0.032	0.011	0.037	0.746
LEG1019	01:01/04N	03:01	44:02	57:04	05:01/03	18:01	04:01	15:01	DRB4	DRB5	02	03:01	0.010	1.00E-05	0.067	0.510	0.072	4.00E-05	0.341	1.00E-05
LEG1021	03:01	32:01	35:03	52:01	12:02	12:03	09:01	15:02	DRB4	DRB5	03:03	06:01	0.019	0.010	0.215	0.003	1.00E-05	0.002	0.062	0.689
LEG1025	03:02	23:01	08:01	14:02	07:02	08:02	03:01	03:02	DRB3	DRB3	02	04:02	0.003	1.00E-05	0.162	0.193	0.413	0.012	0.217	1.00E-05
LEG1028	11:01	24:02	38:01	40:10	04:03	12:03	13:02	15:01	DRB3	DRB5	05:02	06:03	0.016	0.037	2.00E-05	0.010	0.013	0.827	0.091	0.006

Participant HLA genotyping (up to four digits) and ancestry as determined by genotype-dependent admixture analysis (at K = 18). AFR, African; AMR, Admixed American; CSA, Central/South Asian; EAS, East Asian; EUR, European; MDE, Middle Eastern; n.f., not found; OCE, Oceanian; SAS, South Asian.

### Temporal regulation of binding and functional serological responses to aQIV

Each aQIV 2022–2023 influenza vaccine administered to the LEGACY study participants contained four 15 μg doses of the homotrimeric glycoprotein haemagglutinin (HA), raised from A/Victoria/2570/2019 IVR-215, A/Darwin/6/2021 IVR-227, B/Austria/1359417/2021 BVR-26 and B/Phuket/3073/2013 BVR-1B. Participant responses to the vaccine were measured by means of binding Ig ELISA, multiplexed immunoassay (Luminex) and haemagglutination inhibition (HAI) assays, using participant sera obtained before, upon and after vaccination (Fig. 1E and F; Supplementary Figure S1). At the early and late post-vaccination time points there was a significant increase in antigen-specific IgG to the four-vaccine virus strain HAs (Fig. 1E). Multiplexed immunoassaying revealed that the IgG response was largely IgG1 (*P* < 0.05 for all vaccine HAs), whilst IgG3 also contributed towards A/Darwin and A/Victoria responses at the early time point (Supplementary Figure S1; Supplementary Figure S2). There was also a rapid IgM response to A/Darwin and B/Austria at the early time point (*P* < 0.01) which began to wane by the late time point, and a rapid IgA1 response (*P* < 0.01 for all vaccine HAs) which was sustained against the vaccine antigens (*P* < 0.001 for all vaccine HAs) (Supplementary Figure S1). Although not included in the vaccine, there was also an increase in IgG towards both avian HA subtypes H5N1 and H5N8 by the late post-vaccination time point (Supplementary Figure S2; Supplementary Figure S3). Where there was no pre-existing protection, participants seroconverted (HAI ≥40) against all four vaccine antigens (Fig. 1F), and the HAI levels were positively correlated with the antigen-specific IgG levels (Supplementary Figure S4).

To assess how the serological response was associated with the exact day of post-vaccination sampling, we correlated this with the ELISA, multiplexed immunoassay and HAI assay results for the early and late sampling time points (Fig. 1G). At the early time point (days 3–7), the ELISA antigen-specific IgG response, the Luminex IgG1, IgM and IgA1 and the HAI responses were positively correlated with the day of post-vaccination sampling, but this correlation did not persist during the late time point (days 26–42), during which the responses had typically begun to plateau (Fig. 1E–G; Supplementary Figure S1).

Correlating the serological responses with participant characteristics including age, BMI, LN diameter, and exact day of post-vaccination sampling revealed significant positive correlations (*P*_*adj*_ < 0.05) between early and late post-vaccination time point fold-change across multiple serological response metrics as well as early serological responses with the pre-vaccination draining lymph node diameter (Supplementary Table S1). To further investigate the associations between antibody titres pre- and post-vaccination we used Bayesian hierarchical linear modelling to examine the effect of pre-vaccination IgG titres on post-vaccination responses, and the contribution of IgG subtypes to total IgG titres across strains. No significant associations were observed, apart from a positive association between the pre-vaccination IgG titre against the Darwin strain with the post-vaccination titre (Supplementary Figure S5).

### LN cellular composition is both temporally and anatomically regulated

During FNA visits, bright (B)-mode US images were taken of dLNs and ndLNs and measurements taken of their long-axis diameter. Prior to vaccination during steady state, dLN and ndLN diameters averaged 11.4 mm and 11.0 mm, respectively, and did not differ significantly. Post-vaccination, there was a significant increase in dLN diameter to 15.6 mm on average. However, there was no significant detectable change in the ndLN size (Fig. 1H). The post-vaccination dLN size was significantly negatively correlated with the number of days after vaccination at the early time point, but this was not the case for the ndLN (Fig. 1I).

Further to investigating LN size changes, we also assessed the yield of LN cells from the US-guided FNA. Yields were variable, but in the same range as previous studies.^14^^,^^20^ To determine which factors were contributing to this variation, a principal component analysis (PCA) was undertaken. Donor quantitative factors such as height and age, the total cell count after sample processing, the study time points, alongside LN size were considered. The number of days post vaccination contributed significantly to the cell yield. Donor height and weight were also strong contributors to LN cell yield, especially at steady state prior to vaccination (Fig. 1J–L).

### A longitudinal multi-modal scRNA-seq vaccination atlas of axillary dLNs and ndLNs

**Fig. 2:**
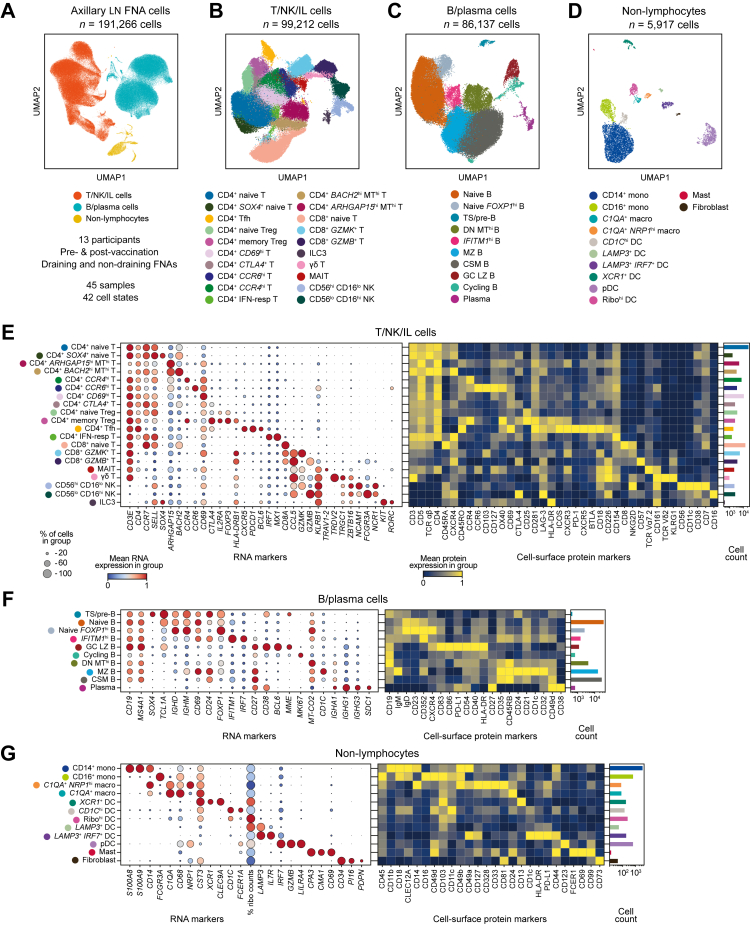
**Characterisation of LN FNA cell populations and cell states identified using single-cell transcriptomics and cell-surface protein expression.** (A) Uniform Manifold Approximation and Projection (UMAP) representation of the three broad cell compartments identified in LN FNA samples pre- and post-vaccination. (B–D) Higher resolution annotation of T/NK/IL and B/plasma cell and non-lymphocyte compartments. CSM, class-switched memory B cell; DC, dendritic cell; DN, double negative B cell; FNA, fine-needle aspirate; GC LZ, germinal centre light zone B cell; hi, high; IFN-resp, interferon-responsive; IL, innate lymphoid; lo, low; macro, macrophage; MAIT, mucosal-associated invariant T; mono, monocyte; MT, mitochondrial genes; MZ, marginal zone B cell; NK, natural killer; pDC, plasmacytoid dendritic cell; Ribo, ribosomal; Tfh, T follicular helper cell; Treg, T regulatory cell; TS, transitional B cell. (E–G) Key RNA marker genes (left), cell-surface protein expression (middle), and cell counts (right) of each cell state per cell compartment.

Single-cell analysis of 45 LN FNA samples was undertaken from the 13 participants who completed the study protocol, resulting in 191,266 high-quality transcriptomes alongside cell-surface protein and T-cell repertoire data. Three main cell compartments were identified, T/natural killer (NK)/innate lymphoid (IL), B/plasma, and non-lymphoid cells, and were subclustered into 42 different cell states: 20 T/NK/IL cell states (*n* = 99,212 cells), 10 B cell and plasma cell states (*n* = 86,137 cells) and 12 non-lymphoid (myeloid and stromal) cell states (*n* = 5917 cells) (Fig. 2A–D; Supplementary Figure S6A). Amongst these, cell states important for initiating and sustaining the serological vaccine reaction within LNs were readily identified (Fig. 2E–G). The identity of these cell states, which included CD4^+^ T follicular helper (Tfh) cells, germinal centre (GC) light zone (LZ) B and cycling B cells, plasma cells and plasmacytoid dendritic cells (pDCs), was confirmed by cell-surface protein marker expression based on antibody-derived tags (Fig. 2E–G). For example, Tfh cells expressed marker transcripts *CD3E*, *CD4*, *CD69*, *CXCR5*, *PDCD1*, and *BCL6*. The same cell state also expressed the protein markers CD3, CD4, CD45RO, PD-1, ICOS, CXCR3, CXCR5, B and T lymphocyte attenuator (BTLA) and CD18 with intermediate CD57 expression suggesting a transitional Tfh cell state^40^ (Fig. 2E). Other notable non-naïve lymphocyte cell states detected included CD8^+^ *GZMK*^+^ T cells, CD8^+^ *GZMB*^+^ T cells and mucosa associated invariant T (MAIT) cells. Non-lymphoid cells were less abundant but highly diverse, particularly amongst dendritic cells which included six different identifiable cell states (Fig. 2D–G). The top 50 differentially expressed genes from each cell state can be referenced in Supplementary Table S2.

To compare the cell states identified in our data with previously published dLN scRNA-seq data derived from a non-adjuvanted influenza vaccination study,^17^ we performed reference mapping of our annotations onto 48,376 T cells from this study (Supplementary Figure S6B). These T cells were obtained from two donors, who were the only participants sampled at time-points analogous to our study (before and 5–7 days after vaccination). The reference mapping demonstrated that the T/NK/IL cell states we annotated were identifiable in this dataset, and vice versa, reference mapping of the Schattgen et al.^17^ annotations demonstrated that their clusters were identifiable in our data as well (Supplementary Fig. S6B and C), suggesting cell state concordance in the dLN across studies. Downstream analyses utilising the Schattgen et al.^17^ data were not performed due to a lack of power given the small number of individuals sampled at relevant post-vaccination time-points.

### Influenza vaccination induces anatomical and temporal-specific differential cellular abundance

**Fig. 3:**
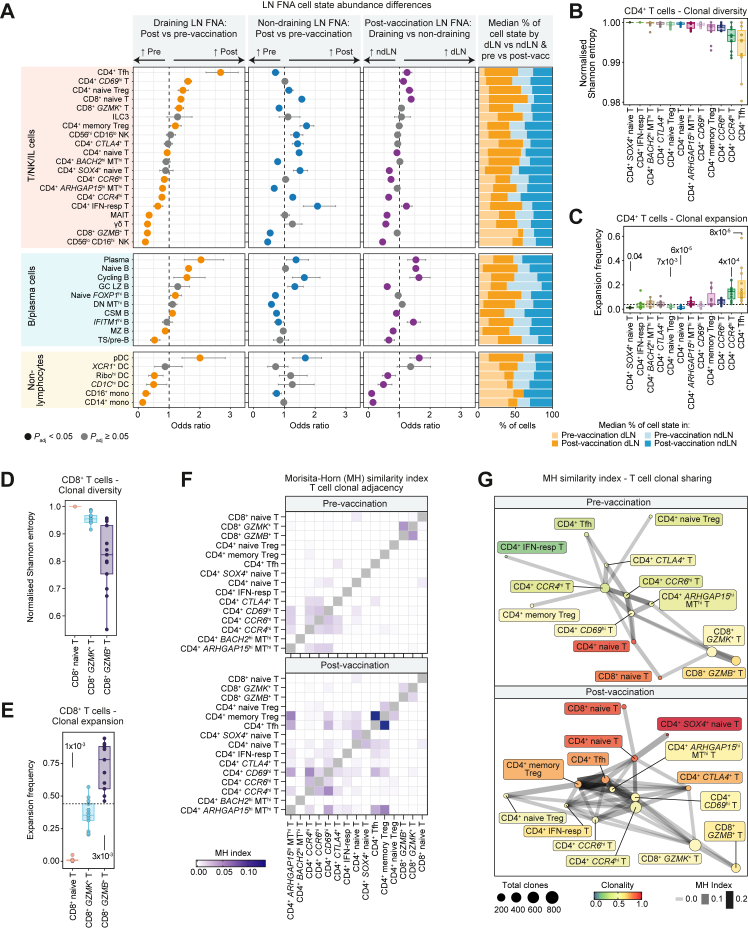
**Adjuvanted influenza vaccine stimulates differential abundance and T-cell clonal dynamic changes across cell states, sample sites and time points.** (A) MASC was used to test abundance differences across cell compartments after influenza vaccination (see Methods for more details). Comparison of pre-vaccination and post-vaccination samples from draining LNs (dLNs; orange; pre-vaccination: *n* = 10, post-vaccination: *n* = 12) and non-draining LNs (ndLNs; blue; pre-vaccination: *n* = 11, post-vaccination: *n* = 12) as well as a post-vaccination comparison of dLNs and ndLNs (purple) represented as odds ratios (OR) are shown. Non-grey dots indicate significant differences (*P*_*adj*_ < 0.05) after Benjamini-Hochberg correction. Error bars show the 95% confidence interval. Median proportion of each cell state by sample site and time point is also represented in a stacked bar plot. (B–E) A total of 80,427 T cells with both transcriptomic and TCR sequences were observed, including 2057 clones (groups of two or more cells sharing TCRs). Normalised Shannon entropy index was estimated for each cell state in the CD4^+^ (B) or CD8^+^ (D) T-cell compartment. Clonotypes included have one alpha and one beta chain. Each dot represents a donor. The expansion frequency was estimated as the proportion of cells in a clone (group with two or more cells) in each cell state in CD4^+^ (C) or CD8^+^ (E) T-cell compartment. The dashed line indicates the mean expansion frequency in that compartment. Wilcox test compared the expansion frequency of each cell type to the mean of the CD4^+^ or CD8^+^ T-cell compartment expansion frequency. (F) Clonal dynamics between cell types pre-vaccination and post-vaccination as indicated by Morisita-Horn (MH) similarity index. (G) Shared clonal networks of pre- and post-vaccination T-cell repertoires after filtering, where edge weights are defined by MH similarity index, node colour denotes clonality, and node size indicates number of clones. CSM, class-switched memory B cell; DC, dendritic cell; DN, double negative B cell; FNA, fine-needle aspirate; GC LZ, germinal centre light zone B cell; hi, high; IFN-resp, interferon-responsive; lo, low; macro, macrophage; MAIT, mucosal-associated invariant T; mono, monocyte; MT, mitochondrial genes; MZ, marginal zone B cell; NK, natural killer; pDC, plasmacytoid dendritic cell; Ribo, ribosomal; Tfh, T follicular helper cell; Treg, T regulatory cell; TS, transitional B cell.

Adjuvants generate a robust immune response in a non-antigen specific manner; the MF59C.1 adjuvant in the vaccine received by the LEGACY study participants is known to support early generation of high-affinity influenza-specific antibodies and circulating Tfh in older adults.^41^ Thus, MF59C.1 potentially affects the dLNs and ndLNs similarly, depending on its pharmacokinetics. Furthermore, cellular infiltration of non-resident memory B cells may be required to support the LN recall response, as shown in mice.^42^ To determine whether an adjuvanted influenza vaccine induces cellular immunity across the lymphatic network, or regionally, we investigated the differential abundance changes in the dLNs and ndLNs before and after vaccination (Fig. 3A; Supplementary Figure S7). To utilise the multiple sample sites per donor as a means of within-donor normalisation, mixed-effects association testing for single-cells (MASC)^33^ was used to test differential abundance between sample sites and time points for each cell state directly, while accounting for weight and number of days post-vaccination as fixed-effect covariates. Overall, the differential abundance changes in the dLN after vaccination were dramatically different to those in the ndLN (Fig. 3A). These results were mirrored in the direct post-vaccination dLN and ndLN comparison. CD4^+^ Tfh cells were significantly higher post-vaccination on the dLN side, but not the ndLN side compared to pre-vaccination. Like our previous findings, naïve B cells, germinal centre and cycling B cells, and CD4^+^ CD69^+^ T cells increased in abundance in dLNs.^20^ Changes in T-cell abundance in ndLNs were amongst cells not typically associated with antigen-specific responses, including CD4^+^ IFN-resp T, MAIT and NK cells. In both dLNs and ndLNs, plasma cells, cycling B cells and pDCs were relatively increased, as were CD4^+^ memory Treg cells, with relative decreases in CD8^+^ *GZMB*^+^ T-cell abundance. This suggested a second, less prominent but measurable global effect of aQIV immunisation on the lymphatic system, that could potentially be attributed to the adjuvant, with highly abundant vaccine-responsive cell types, e.g., CD4^+^ Tfh, diluting this effect in the dLN. The median proportion of each cell state at each sample site and time point further illustrates these findings (Fig. 3A).

Given the abundance differences in cell states associated with the humoural immune response, we assessed the correlation between the pre- and post-vaccination proportions of total B cells and Tfh cells in the dLN and ndLN relative to the early and late post-vaccination time point fold-change in antibody titres (Appendix A, Appendix A). Whilst no correlations were statistically significant, there was a trend towards positive correlations in the pre- and post-vaccination dLN B-cell proportions with multiple antibody isotypes and viral strains, whereas for the ndLN trends towards both positive and negative correlations were observed. For the Tfh cells there were trends towards more isotype-specific correlations by time-point and LN site (Appendix A, Appendix A).

### Vaccination-induced early changes in LN T-cell repertoire

We next investigated the dynamic changes in the T-cell repertoire upon vaccination to further elucidate the relationships between T cells. Using Immcantation, IgBlast and scRepertoire framework for V(D)J gene assignment and productive alpha-beta paired clonotype filtering, clones were identified using TCR genes and the CDR3 nucleotides on both alpha and beta chains. Normalised Shannon entropy index revealed a relative drop in the diversity of CD4^+^ Tfh and CD4^+^ *CCR4*^hi^ T cells, and a corresponding increase in the clonal expansion frequency of these cell states compared to the median expansion frequency of CD4^+^ T cells (*P* ≤ 4 × 10^−4^) (Fig. 3B and C). This suggests that the increase of CD4^+^ Tfh and CD4^+^ *CCR4*^hi^ T cells in LNs is not only a reflection of increased recruitment from the circulation, but also of expansion and differentiation. Within the CD8^+^ T-cell compartment, the normalised Shannon entropy index decreased for the CD8^+^ *GZMB*^+^ T cells, with a concomitant increase in the clonal expansion of these cells (*P* = 3 × 10^−3^) (Fig. 3D and E).

The Morisita-Horn similarity index was used to compare the T-cell immune repertoire before and after vaccination among different T-cell states. Post-vaccination clonal dynamics were found to be more active than pre-vaccination, especially in the CD4^+^ T-cell compartment (Fig. 3F and G). CD4^+^ *CCR4*^hi^ T cells, which showed small but significant changes in abundance bilaterally, had heavily expanded TCRs and after vaccination this cell type remained a key “hub”, showing the most clonal connections to other cells and an increased clonal link to CD4^+^ *CD69*^hi^ T cells post vaccination (Fig. 3G).

### Influenza vaccination stimulates gene expression hubs associated with dLNs but not ndLNs

**Fig. 4:**
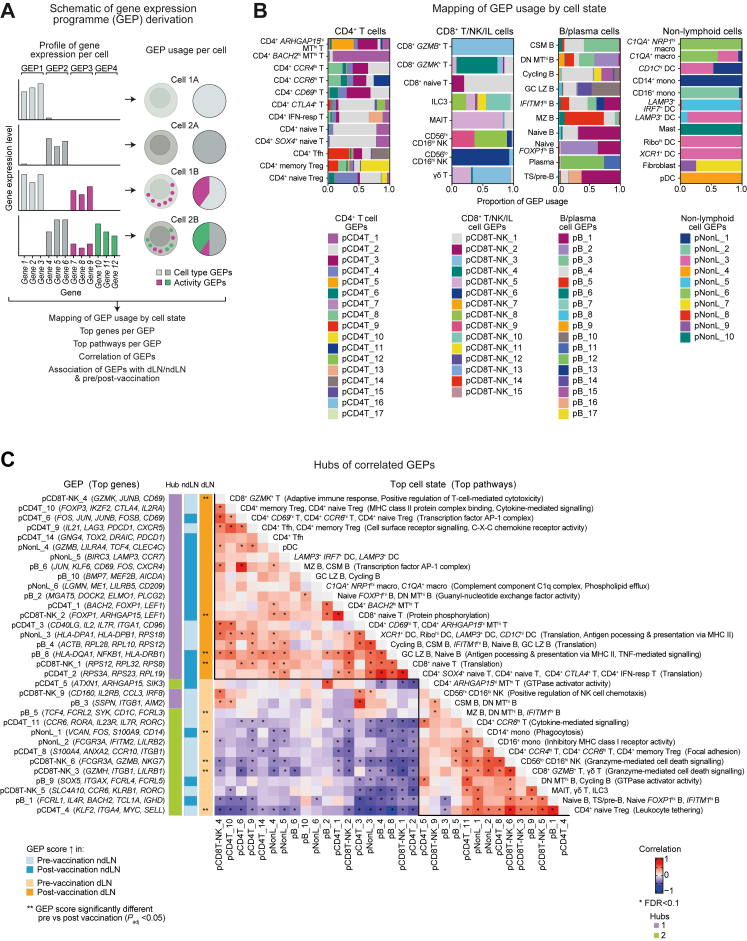
**Differential gene expression hubs associated with influenza vaccination.** (A) Schematic of cNMF analysis identifying gene expression programmes (GEPs), their characterisation, correlation between significant GEPs, and association with sample site and time points (adapted from https://github.com/dylkot/cNMF). (B) Mapping of GEP usage by cell state across CD4^+^ T (1–17), CD8^+^ T/NK/IL (1–15), B/plasma (1–17), and non-lymphoid cell (non-L) (1–10) compartments. Different contributions are shown in the top row and the numbered GEPs in the bottom row. (C) Correlations of significant GEPs with annotations of top genes, pathways and cell types. ∗ indicates significantly correlated GEP pairs by Pearson correlation (FDR <0.1). Solid lines demarcate hubs of highly correlated GEPs. Hub annotation indicates two clusters from hierarchical clustering. dLN and ndLN annotations denote which GEPs have greater expression at each sample site and time point. ∗∗ indicates the GEPs that have significantly different expression pre-vs post-vaccination (*P*_*adj*_ < 0.05). CSM, class-switched memory B cell; DC, dendritic cell; DN, double negative B cell; FNA, fine-needle aspirate; GC LZ, germinal centre light zone B cell; hi, high; IFN-resp, interferon-responsive; IL, innate lymphoid; lo, low; macro, macrophage; MAIT, mucosal-associated invariant T; mono, monocyte; MT, mitochondrial genes; MZ, marginal zone B cell; NK, natural killer; pDC, plasmacytoid dendritic cell; Ribo, ribosomal; Tfh, T follicular helper cell; Treg, T regulatory cell; TS, transitional B cell.

To further investigate the heterogeneous immune response after influenza vaccination, we utilised consensus non-negative matrix factorisation (cNMF) to identify gene expression programmes (GEPs) within cell types.^29^ Considering that a cell's gene expression can reflect its cell type concurrently alongside its metabolic and functional state, any individual cell can comprise multiple GEPs (Fig. 4A). After identifying the GEPs of each broad compartment (CD4^+^ T cells numbered 1–17, CD8^+^ T/NK/IL cells numbered 1–15, B/plasma cells numbered 1–17, and non-lymphocytes numbered 1–10), we assessed the contribution of each GEP to each cell type (Fig. 4B) and the top genes/pathways to each GEP (Fig. 4C). For instance, CD4^+^ Tfh cells comprised of multiple GEPs including GEPs pCD4T_9 and pCD4T_14 that identified the cell state with genes such as *PDCD1* and *CXCR5*, as well as shared GEPs pCD4T_2 and pCD4T_3 that mapped to many other CD4^+^ T cell states and included genes such as *IL7R*, *CD96*, and ribosomal genes.

To identify GEPs that have significantly different distributions between the draining and non-draining LN and by sampling time points, Wasserstein distances were calculated and compared to the null distribution (the median of the permuted scores). Significant GEPs were identified as those with q-values <0.05, where the q-value is defined as the threshold that minimises the false discovery rate (FDR). Significant GEPs were then further correlated with each other to identify hubs of related biological processes. We derived two hubs using hierarchical clustering (Fig. 4C). We assessed how the individual GEPs changed pre- and post-vaccination in the dLNs and ndLNs. GEPs associated with hub 1 had a higher median expression post-vaccination in the dLN but not the ndLN. Post-vaccination GEPs in hub 1 included genes associated with CD4^+^ Tfh and CD4^+^ memory Treg, pDC and cycling B cells. Notably, across different immune cell types, this hub included GEPs (pCD8T-NK_4, pCD4T_6, and pB_6) denoted by the expression of immune activation genes (*JUN*, *JUNB*, *FOS*, and/or *CD69*), consistent with a MAST-based differential gene expression analysis that identified many of these genes as being significantly increased post-vaccination across several LN immune cell subtypes (Supplementary Table S3). Notably, comparing the differentially expressed pathways between the dLN and ndLN, interferon response pathways were most consistently increased in the dLN across adaptive immune cell types (Supplementary Table S4). GEPs associated with hub 2 had increased median expression pre-vaccination in the dLN but not the ndLN.

## Discussion

A key objective to achieve global research equity is to determine the influence of ancestral diversity on vaccine-induced immunity to respiratory pathogens such as influenza; this has been poorly addressed in the past in genetic research and in vaccine development.^43^, ^44^, ^45^, ^46^, ^47^ Here we present an extensive anatomical, proteomic, genomic, genetic and kinetically dynamic investigation of the human organ that is critical to generating vaccine immunity, the lymph node, in a diverse cohort. This study demonstrated that the early (1 week) lymphatic response to IM immunisation with aQIV was tightly temporally, genetically and regionally regulated. This response pattern was broadly consistent across young adults receiving aQIV, which contains MF59C.1, and was not strongly influenced by ancestral diversity, despite cohort-wide differences in genotype and HLA type. Early cellular events in the draining lymph node were significantly different from the non-draining lymph node, indicative of a regionally activated antigen-driven response coupled with a generalised inflammatory response that was observed bilaterally.

Using US to serially track responses, immunisation into the deltoid muscle was associated with a fast onset of regional swelling in the dLNs only, with an indication that this swelling might resolve equally rapidly. This early swelling was accompanied by an influx of adaptive immune cells including CD4^+^ Tfh and B cells, with a drop in clonal diversity, indicating that T-cell clonal expansion is an active process within the dLN. Regional LN swelling appeared to be highly sensitive to immunisation, suggesting that this may play a role in the biological mechanisms underlying vaccine responsiveness. This finding builds on recent work on later-stage cellular events in the dLN with non-adjuvanted influenza vaccine.^17^ We demonstrated that early dLN swelling can be directly linked to cellular infiltration and expansion, consisting of multiple vaccine-responsive cell types dominated by CD4^+^ Tfh, in the first few days, post vaccination. Conversely, cellular dynamics of the ndLN have not been previously studied by FNA and single-cell multi-omics, although two flow cytometric studies include the ndLN after COVID-19 or seasonal influenza immunisation.^48^^,^^49^ Our data show that whilst some non-specific changes occur in the ndLN, the coordinated swelling and adaptive immune cell increase are restricted to draining lymphatic tissue in the week post vaccination.

The cohort in this study had several HLA alleles that have been rarely described in single-cell/pMHC atlasing but are nonetheless common globally. The paucity of HLA peptide binding data for HLA alleles in non-European ancestry populations, such as those we identified, presents a substantial gap in our knowledge, which studies such as this are needed to address, as reviewed in Ref.^43^ This knowledge gap has consequences for rational vaccine design. Generation of novel antigens for vaccine discovery are increasingly utilising computational methods and prediction of HLA epitope binding via machine learning.^50^, ^51^, ^52^ This approach is critically dependent on the quality and breadth of databases. Machine learning models are currently inherently biased as most input data are derived from individuals of European ancestry, with a lack of HLA diversity that fails to reflect the global population.^53^ By studying diverse cohorts, and making HLA data publicly available, machine learning models will ultimately become less biased supporting a tailored and equitable approach to antigen generation.

This study pinpointed the early induction of CD4^+^ Tfh as pivotal to the lymph node response to aQIV immunisation in the draining tissue at all levels of analysis conducted (genetic, proteomic and gene expression). CD4^+^ Tfh are specialised T helper cells that select B cells for survival and expansion in the light zone of the germinal centre with a pivotal role in the size and quality of the antibody response. The unadjuvanted cell-based influenza vaccine induces and matures the Tfh response in the lymph node in young adults.^17^ CD4^+^ *CCR4*^hi^ T cells were the second subset where a drop in clonal diversity and increase in clonal expansion in the draining LN was seen. The role of CD4^+^ *CCR4*^hi^ T cells in vaccine responses has not previously been identified in humans *in vivo*. CCR4 affects T-cell LN homing and T regulatory cell recruitment to the site of immunisation in mice and CCR4 antagonists target T regulatory and Th2 cells with adjuvant activity *in silico*.^54^, ^55^, ^56^

The seasonal influenza vaccine used in this study, aQIV, contains MF59C.1, a squalene containing compound. Young adults typically do not receive adjuvanted seasonal influenza vaccines; however in an influenza pandemic setting, there is a conceivable advantage to their use, and adjuvanted avian influenza vaccines have been developed.^57^ The aQIV induced a broadly cross-reactive response to haemagglutinins contained within the vaccine and to avian influenza subtypes H5N1 and H5N8. Although we did not have an unadjuvanted vaccine to directly compare, the rapid induction of the Tfh response in the draining lymph node coupled with robust serological responses indicates this vaccine to be immunogenic in the setting of ancestral diversity.

This study had limitations; the deep genetic and immunophenotyping conducted on LN cells was only possible in the relatively small sample size of this cohort. The lack of TCR-peptide-MHC diversity in publicly available reference data greatly limits our capacity to accurately demonstrate antigen specificity. Together, this restricted the utility of additional analyses including testing expanded TCRs for antigen-specificity. Nonetheless, previous studies of LN responses in humans have typically included between 5 and 15 individuals and we directly compared our data with a publicly available dataset from LNs sampled at relevant time points.^14^, ^15^, ^16^, ^17^, ^18^ In addition, data from our study compared the bilateral LN immunisation response coupled with a detailed ancestral analysis. Whilst our data include representation of self-reported ethnicity, we did not collect data on other relevant social constructs such as socio-economic status as the impact of these would not have been testable in this cohort size. We did however control for time resident in the UK in the inclusion criteria. The publicly accessible dataset that we have generated will allow future meta-analyses to test new research questions across several such studies, for instance to conduct well-powered investigations of the similarities and differences in vaccine responses by ancestral group or to generate medications or vaccines that are HLA-targeted. This inclusive approach for minoritised populations will support the achievement of global health equity.^58^

Here we have shown the cellular and genetic determinants of the regionally and temporarily defined early lymphatic response to intramuscular immunisation with an adjuvanted seasonal influenza vaccine, in an ancestrally diverse cohort of young adults. The early vaccine response gene signature to aQIV could be detected in the dLN. This vaccine-specific gene signature mapped directly to the LN response to aQIV in healthy young adults with diverse ancestry and was associated with robust vaccine immunogenicity and could serve as benchmark for optimal early immune responses. The dataset arising contributes towards addressing gaps in reference material underpinning basic and translational discoveries in human immunology and vaccine development.

## Contributors

J.H.Y. Siu: Data curation, Formal analysis, Investigation, Methodology, Project Administration, Supervision, Visualisation, Writing—original draft, Writing—review & editing; S. Coelho: Data curation, Investigation, Methodology, Project administration; A. Palomeras: Data curation, Methodology, Project administration; S. Belij-Rammerstorfer: Methodology, Investigation, Formal analysis; N. Barman: Methodology, Investigation, Formal analysis; C.H. Lee: Investigation, Formal analysis; T. Strobel: Investigation, Formal analysis; C.J. Thorpe: Investigation, Formal analysis; C. Kaur: Data curation, Investigation; T. Cole: Project administration; N. Remmert: Formal analysis, Visualisation; J. Fowler: Investigation; S. Pledger: Investigation; K.B. Dooley: Investigation; T. Chan: Investigation; K. Höschler; Methodology, Investigation, Formal analysis. M. Zambon; Methodology, Investigation. D. Opoka: Investigation; T. Szommer: Data curation, Project administration; S.J. Kim: Investigation; V. Kumar: Project administration; S. Vanderslott: Funding acquisition, Methodology; P. Kaleebu: Conceptualisation, Supervision, Funding acquisition; A. Milicic: Funding acquisition, Writing—review & editing; D.B. Palmer: Funding acquisition, Writing—review & editing; T. Lambe: Conceptualisation, Funding acquisition, Resources, Supervision; B.D. Marsden: Data curation, Funding acquisition, Project administration, Supervision; H. Koohy: Funding acquisition, Supervision, Writing—review & editing; M. Coles: Conceptualisation, Funding acquisition, Resources, Supervision, Writing—review & editing; C.A. Dendrou: Conceptualisation, Formal analysis, Funding acquisition, Investigation, Methodology, Project administration, Resources, Supervision, Visualisation, Writing—original draft, Writing—review & editing; K.M. Pollock: Conceptualisation, Funding acquisition, Investigation, Methodology, Project administration, Resources, Supervision, Writing—original draft, Writing—review & editing. J.H.Y. Siu, C.A. Dendrou and K.M. Pollock accessed and verified the underlying data. All authors read and approved the final version of the manuscript. J.H.Y. Siu, C.A. Dendrou and K.M. Pollock were responsible for the decision to submit the manuscript.

## Data sharing statement

De-identified individual participant data that underlie the results presented in this article are available through the accompanying supplementary data and through the source data that can be obtained via Zenodo (https://doi.org/10.5281/zenodo.15745083). All code associated with the study is also available via Zenodo (https://doi.org/10.5281/zenodo.15783552). User-friendly access to the single-cell data from this study is enabled by CELLxGENE (https://cellxgene.cziscience.com/collections/131c68c1-c457-4e3b-88bc-92c4e43ed680). Requests for data sharing should be directed to the corresponding authors.

## Declaration of interests

KMP has received research funding support from Horizon 2020, the NIHR, and The Sir Joseph Hotung Charitable Settlement outside the submitted work. KMP has received honoraria from CSL Seqirus for speaking, travel support from CZI and has participated in Moderna data safety monitoring boards. KMP has a role on the British HIV Association immunisation guidelines writing committee, the UK Clinical Vaccine Network and was on the UK NIHR COVID-19 Chief Investigators group. TL is named as an inventor on a patent application for a vaccine against SARS CoV-2. All other authors declare no conflicts of interest.

## References

[1] Iuliano A.D., Roguski K.M., Chang H.H. (2018). Global seasonal Influenza-associated mortality collaborator network. Estimates of global seasonal influenza-associated respiratory mortality: a modelling study. Lancet.

[2] Auton A., Brooks L.D., Durbin R.M., The 1000 Genomes Project Consortium (2015). A global reference for human genetic variation. Nature.

[3] Coussens A.K., Wilkinson R.J., Nikolayevskyy V. (2013). Ethnic variation in inflammatory profile in tuberculosis. PLoS Pathog.

[4] Mostafavi S., Yoshida H., Moodley D. (2016). Parsing the interferon transcriptional network and its disease associations. Cell.

[5] Zhao H., Harris R.J., Ellis J., Pebody R.G. (2015). Ethnicity, deprivation and mortality due to 2009 pandemic influenza A(H1N1) in England during the 2009/2010 pandemic and the first post-pandemic season. Epidemiol Infect.

[6] Davidson J., Banerjee A., Mathur R. (2021). Ethnic differences in the incidence of clinically diagnosed influenza: an England population-based cohort study 2008–2018. Wellcome Open Res.

[7] Chandrasekhar R., Sloan C., Mitchel E. (2017). Social determinants of influenza hospitalization in the United States. Influenza Other Respir Viruses.

[8] Sze S., Pan D., Nevill C.R. (2020). Ethnicity and clinical outcomes in COVID-19: a systematic review and meta-analysis. EClinicalMedicine.

[9] Mathur R., Rentsch C.T., Morton C.E. (2021). Ethnic differences in SARS-CoV-2 infection and COVID-19-related hospitalisation, intensive care unit admission, and death in 17 million adults in England: an observational cohort study using the OpenSAFELY platform. Lancet.

[10] Havenar-Daughton C., Newton I.G., Zare S.Y. (2020). Normal human lymph node T follicular helper cells and germinal center B cells accessed via fine needle aspirations. J Immunol Methods.

[11] Liang F., Lindgren G., Sandgren K.J. (2017). Vaccine priming is restricted to draining lymph nodes and controlled by adjuvant-mediated antigen uptake. Sci Transl Med.

[12] Vella L.A., Buggert M., Manne S. (2019). T follicular helper cells in human efferent lymph retain lymphoid characteristics. J Clin Invest.

[13] Cole M.E., Saeed Z., Shaw A.T. (2019). Responses to quadrivalent influenza vaccine reveal distinct circulating CD4+CXCR5+ T cell subsets in men living with HIV. Sci Rep.

[14] Turner J.S., Zhou J.Q., Han J. (2020). Human germinal centres engage memory and naive B cells after influenza vaccination. Nature.

[15] Turner J.S., O'Halloran J.A., Kalaidina E. (2021). SARS-CoV-2 mRNA vaccines induce persistent human germinal centre responses. Nature.

[16] Mudd P.A., Minervina A.A., Pogorelyy M.V. (2022). SARS-CoV-2 mRNA vaccination elicits a robust and persistent T follicular helper cell response in humans. Cell.

[17] Schattgen S.A., Turner J.S., Ghonim M.A. (2024). Influenza vaccination stimulates maturation of the human T follicular helper cell response. Nat Immunol.

[18] Borcherding N., Kim W., Quinn M. (2024). CD4+ T cells exhibit distinct transcriptional phenotypes in the lymph nodes and blood following mRNA vaccination in humans. Nat Immunol.

[19] Pollock K.M., Dendrou C.A. (2023). An experimental medicine study of seasonal influenza vaccination responses in Lymph nodE single-cell Genomics in AnCestrY (LEGACY01) [Internet]. Protocolsi.o.

[20] Day S., Kaur C., Cheeseman H.M. (2022). Comparison of blood and lymph node cells after intramuscular injection with HIV envelope immunogens. Front Immunol.

[21] Abi-Rached L., Gouret P., Yeh J.H., Di Cristofaro J., PontarottiP P.C., Paganini J. (2018). Immune diversity sheds light on missing variation in worldwide genetic diversity panels. PLoS One.

[22] Vita R., Mahajan S., Overton J.A. (2019). The immune epitope database (IEDB): 2018 update. Nucleic Acids Res.

[23] Bergstrom A., McCarthy S.A., Hui R. (2020). Insights into human genetic variation and population history from 929 diverse genomes. Science.

[24] Ziyatdinov A., Torres J., Alegre-Diaz J. (2023). Genotyping, sequencing and analysis of 140,000 adults from Mexico City. Nature.

[25] Gelman A., Jakulin A., Pittau M.G., Su Y.S. (2008). A weakly informative default prior distribution for logistic and other regression models. Ann Appl Stat.

[26] Gabry J., Simpson D., Vehtari A., Betancourt M., Gelman A. (2019). Visualization in Bayesian workflow. J R Stat Soc Ser A Stat Soc.

[27] Bürkner P.C. (2017). brms: an R package for Bayesian multilevel models using Stan. J Stat Softw.

[28] Bürkner P.C. (2018). Advanced Bayesian multilevel modeling with the R package brms. R Journal.

[29] Gabry J., Češnovar R., Johnson A. Cmdstanr: r interface to ‘CmdStan’. R package version 0.9.0. https://discourse.mc-stan.org.

[30] Carpenter B., Gelman A., Hoffman M.D. (2017). Stan: a probabilistic programming language. J Stat Softw.

[31] Curion F., Rich-Griffin C., Agarwal D. (2024). Panpipes: a pipeline for multiomic single-cell and spatial transcriptomic data analysis. Genome Biol.

[32] Wolock S.L., Lopez R., Klein A.M. (2019). Scrublet: computational identification of cell doublets in single-cell transcriptomic data. Cell Syst.

[33] Fonseka C.Y., Rao D.A., Teslovich N.C. (2018). Mixed-effects association of single cells identifies an expanded effector CD4+ T cell subset in rheumatoid arthritis. Sci Transl Med.

[34] Aran D., Looney A.P., Liu L. (2019). Reference-based analysis of lung single-cell sequencing reveals a transitional profibrotic macrophage. Nat Immunol.

[35] Kotliar D., Veres A., Nagy M.A. (2019). Identifying gene expression programs of cell-type identity and cellular activity with single-cell RNA-Seq. Elife.

[36] Finak G., McDavid A., Yajima M. (2015). MAST: a flexible statistical framework for assessing transcriptional changes and characterizing heterogeneity in single-cell RNA sequencing data. Genome Biol.

[37] Korotkevich G., Sukhov V., Budin N., Shpak B., Artyomov M.N., Sergushichev A. (2021). Fast gene set enrichment analysis. BioRxiv.

[38] Gu Z., Eils R., Schlesner M. (2016). Complex heatmaps reveal patterns and correlations in multidimensional genomic data. Bioinformatics.

[39] Bredikhin D., Kats I., Stegle O. (2022). MUON: multimodal omics analysis framework. Genome Biol.

[40] Padhan K., Moysi E., Noto A. (2021). Acquisition of optimal TFH cell function is defined by specific molecular, positional, and TCR dynamic signatures. Proc Natl Acad Sci U S A.

[41] Li A.P.Y., Cohen C.A., Leung N.H.L. (2021). Immunogenicity of standard, high-dose, MF59-adjuvanted, and recombinant-HA seasonal influenza vaccination in older adults. NPJ Vaccines.

[42] Mesin L., Schiepers A., Ersching J. (2020). Restricted clonality and limited germinal center reentry characterize memory B cell reactivation by boosting. Cell.

[43] Cerdeña J.P., Grubbs V., Non A.L. (2022). Racialising genetic risk: assumptions, realities, and recommendations. Lancet.

[44] Atutornu J., Milne R., Costa A., Patch C., Middleton A. (2022). Towards equitable and trustworthy genomics research. EBioMedicine.

[45] Moxon E.R., Siegrist C.A. (2011). The next decade of vaccines: societal and scientific challenges. Lancet.

[46] Hudson D., Fernandes R.A., Basham M., Ogg G., Koohy H. (2023). Can we predict T cell specificity with digital biology and machine learning?. Nat Rev Immunol.

[47] Olivier T., Haslam A., Tuia J., Prasad V. (2023). Eligibility for human leukocyte antigen-based therapeutics by race and ethnicity. JAMA Netw Open.

[48] Lederer K., Bettini E., Parvathaneni K. (2022). Germinal center responses to SARS-CoV-2 mRNA vaccines in healthy and immunocompromised individuals. Cell.

[49] Law H., Mach M., Howe A. (2022). Early expansion of CD38+ICOS+ GC Tfh in draining lymph nodes during influenza vaccination immune response. iScience.

[50] Pollock K.M., Cheeseman H.M., McFarlane L.R. (2025). Experimental medicine study with stabilised native-like HIV-1 Env immunogens drives long-term antibody responses, but lacks neutralising breadth. EBioMedicine.

[51] Wohlwend J., Nathan A., Shalon N. (2025). Deep learning enhances the prediction of HLA class I-presented CD8+ T cell epitopes in foreign pathogens. Nat Mach Intell.

[52] Bravi B. (2024). Development and use of machine learning algorithms in vaccine target selection. NPJ Vaccines.

[53] Conev A., Fasoulis R., Hall-Swan S., Ferreira R., Kavraki L.E. (2024). HLAEquity: examining biases in pan-allele peptide-HLA binding predictors. iScience.

[54] Palchevskiy V., Xue Y.Y., Kern R. (2019). CCR4 expression on host T cells is a driver for alloreactive responses and lung rejection. JCI Insight.

[55] Yamamoto S., Matsuo K., Nagakubo D. (2018). A CCR4 antagonist enhances DC activation and homing to the regional lymph node and shows potent vaccine adjuvant activity through the inhibition of regulatory T-cell recruitment. J Pharmacol Sci.

[56] Bayry J., Tchilian E.Z., Davies M.N. (2008). In silico identified CCR4 antagonists target regulatory T cells and exert adjuvant activity in vaccination. Proc Natl Acad Sci U S A.

[57] European Medicines Agency Vaccines for pandemic influenza [Internet]. https://www.ema.europa.eu/en/human-regulatory-overview/public-health-threats/pandemic-influenza/vaccines-pandemic-influenza.

[58] Chew M., Samuel D., Mullanc Z., Kleinert S., Lancet Group for Racial Equity (GRacE) (2024). The Lancet Group's new guidance to authors on reporting race and ethnicity. Lancet.

