# Supplementary material for: Early lymph node T follicular helper cell signalling hub drives influenza vaccine response in an ancestrally diverse cohort
Source: eBioMedicine. 2025 Nov 29;122:106036. doi: 10.1016/j.ebiom.2025.106036 (PMC12703867; doi:10.1016/j.ebiom.2025.106036)
Supplement: Supplementary Figure [file mmc5.pdf]

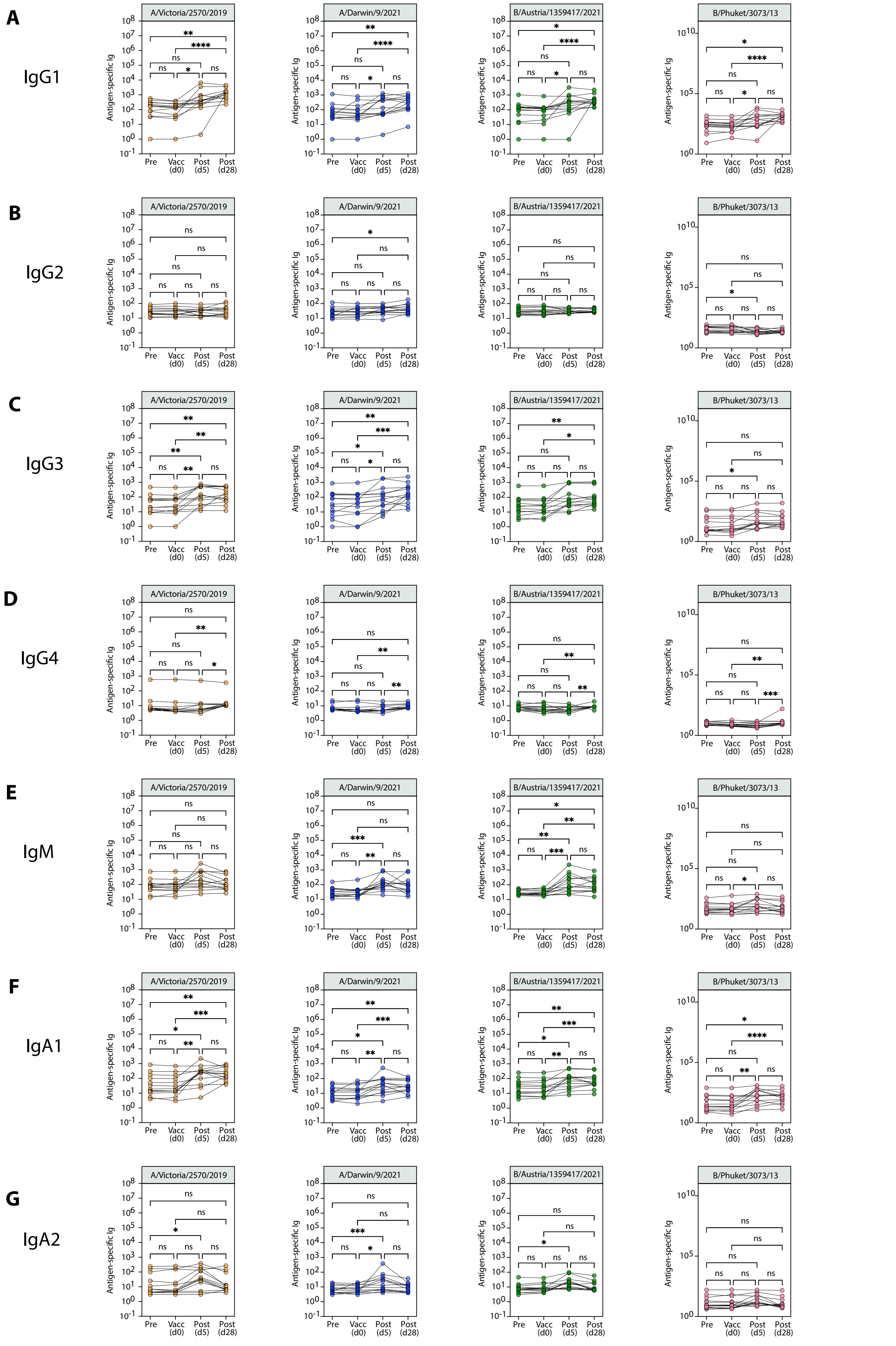


**Figure S1. Detection of antigen-specific antibodies against influenza strains used in the 2022/23 vaccine.** Detection of antigen-specific antibodies - **(A)** IgG1, **(B)** IgG2, **(C)** IgG3, **(D)** IgG4, **(E)** IgM, **(F)** IgA1 and **(G)** IgA2 - by Luminex assay at various timepoints (pre: pre-vaccination, vacc: day of vaccination, post (d5): median five days post-vaccination, post (d28): median 28 days post-vaccination). ns = non-significant; * = P<0.05; ** = P<0.01; *** = P<0.001; **** = P<0.0001.

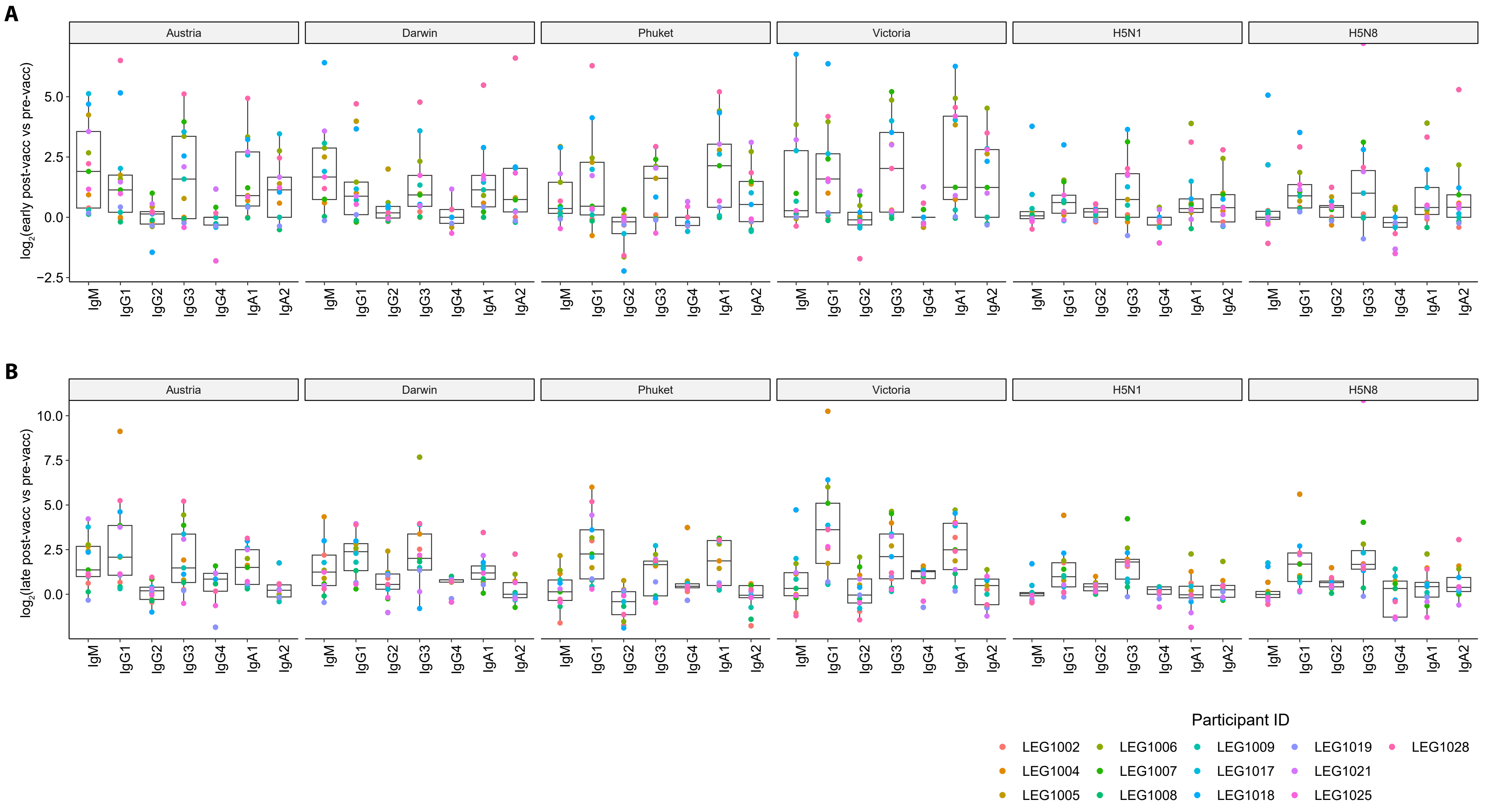


**Figure S2. Fold change in antigen-specific antibodies against influenza strains used in the 2022/23 vaccine and strains H5N1 and H5N8, by vaccination time-point.**

Fold change in antigen-specific antibody (IgM, IgG1, IgG2, IgG3, IgG4, IgA1, IgA2) responses, comparing early (A) or late (B) post-vaccination levels to the pre-vaccination levels. vacc, vaccination.

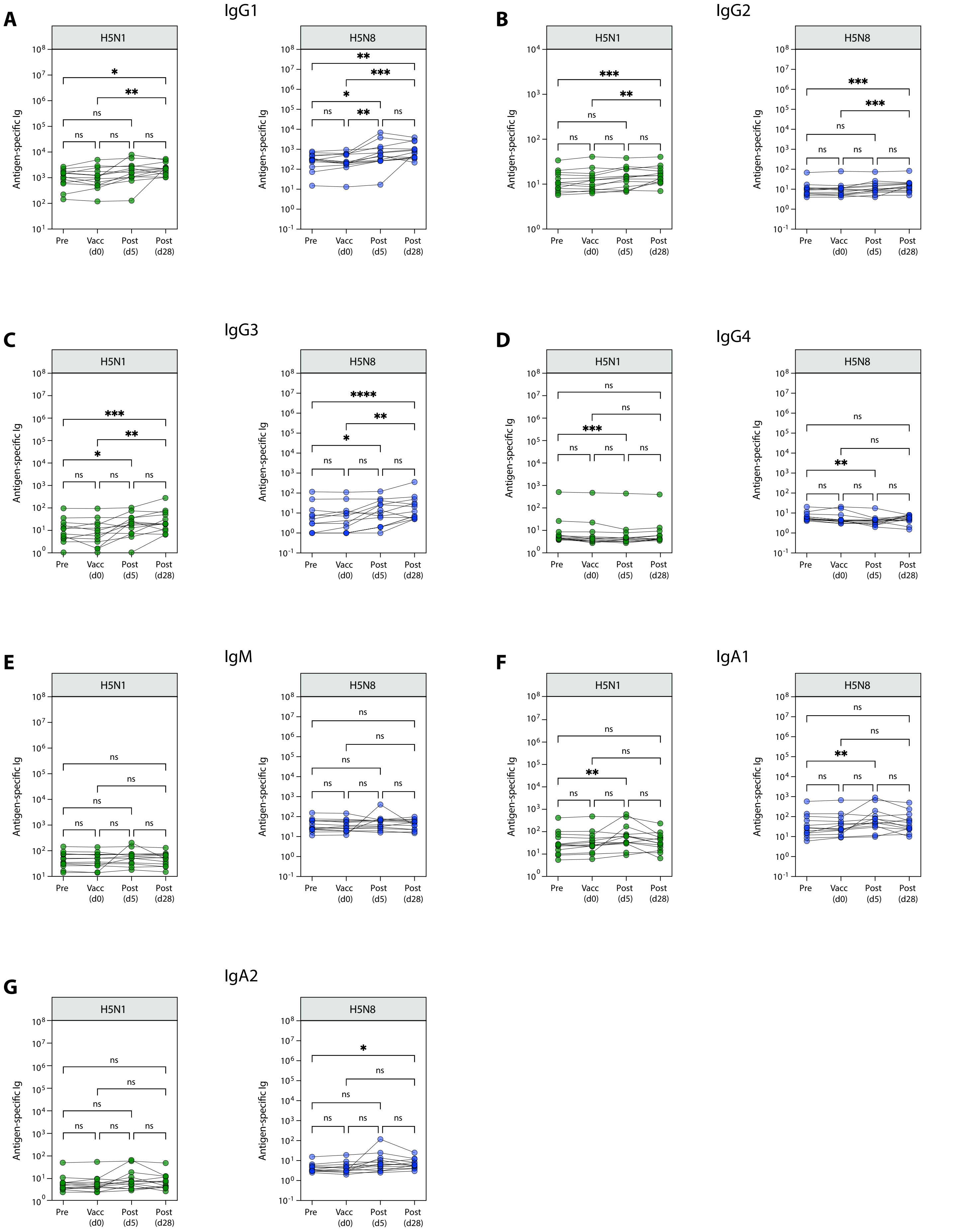


**Figure S3. Detection of antigen-specific antibodies against influenza strains H5N1 and H5N8.**
Detection of antigen-specific antibodies - **(A)** IgG1, **(B)** IgG2, **(C)** IgG3, **(D)** IgG4, **(E)** IgM, **(F)** IgA1 and **(G)** IgA2 - by Luminex assay at various timepoints (pre: pre-vaccination, vacc: day of vaccination, post (d5): median five days post-vaccination, post (d28): median 28 days post-vaccination). ns = non-significant;
* = P<0.05; ** = P<0.01; *** = P<0.001; **** = P<0.0001.

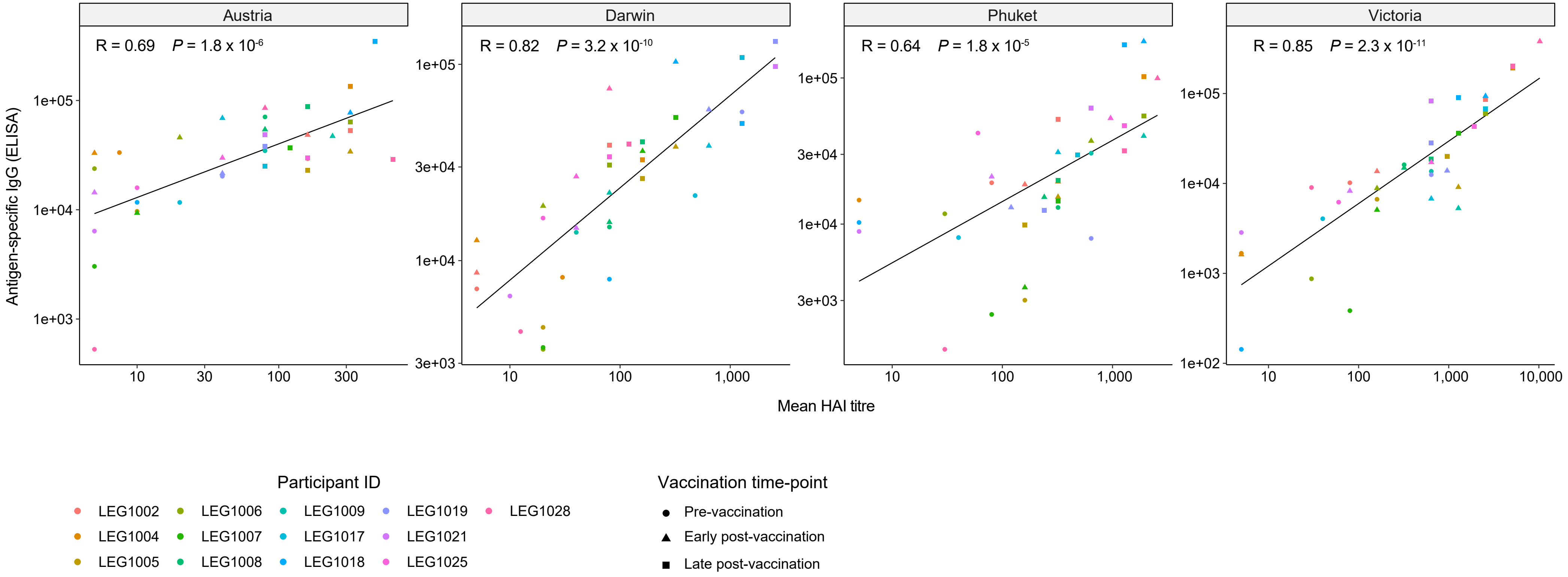


**Figure S4. Correlation analysis of antigen-specific IgG antibodies (against influenza strains used in the 2022/23 vaccine) relative to HAI titre.** Pearson correlation analysis of antigen-specific IgG antibodies detected by ELISA relative to mean HAI titre. R is the correlation coefficient; P is the P-value.

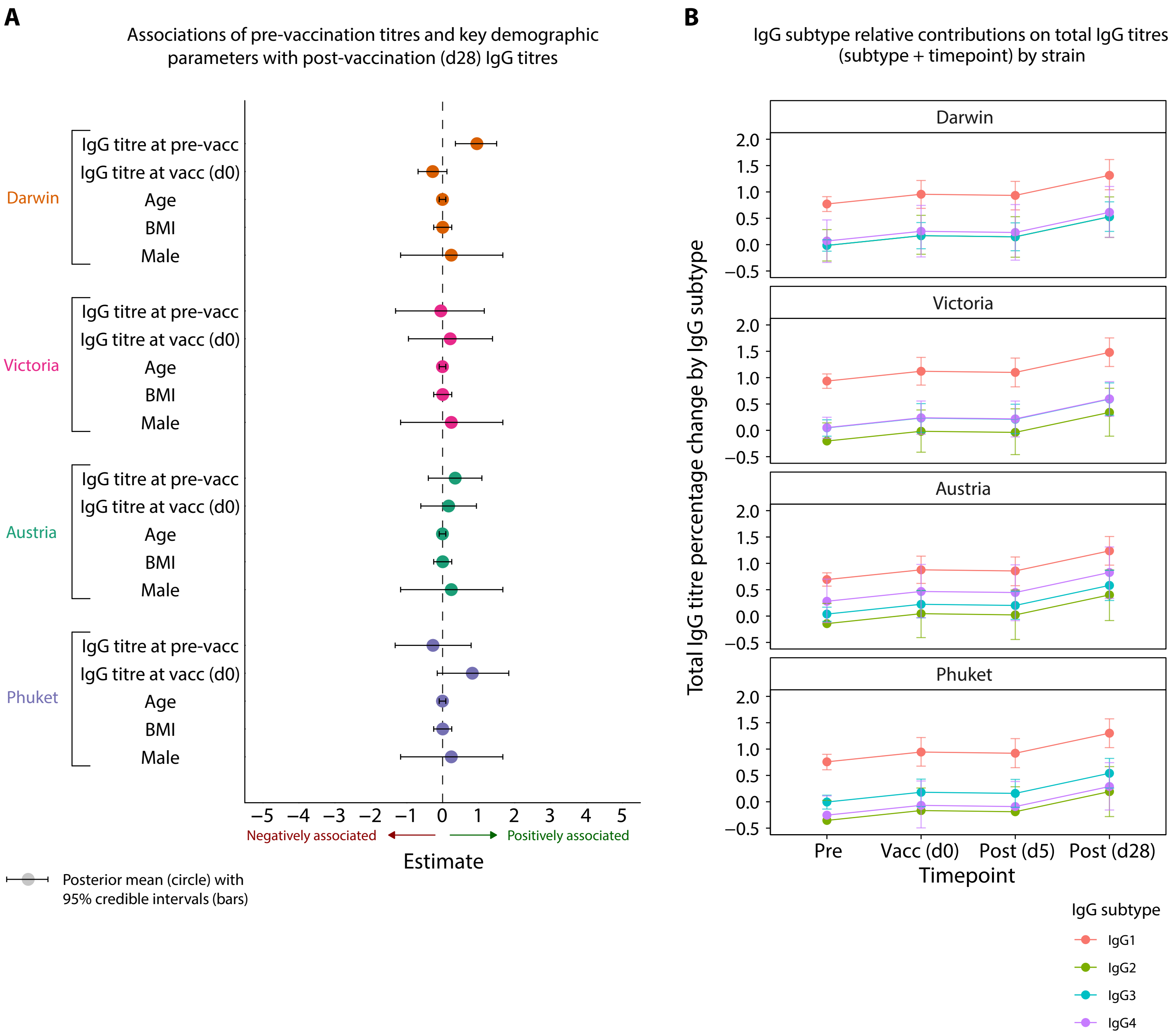


**Figure S5. Bayesian analysis of pre- and post-vaccination antibody titres.**
Bayesian hierarchical linear modelling was used to examine two relationships, **(A)** the effect of pre-vaccination IgG titres on post-vaccination responses, and **(B)** the contribution of IgG subtypes to total IgG titres across strains.

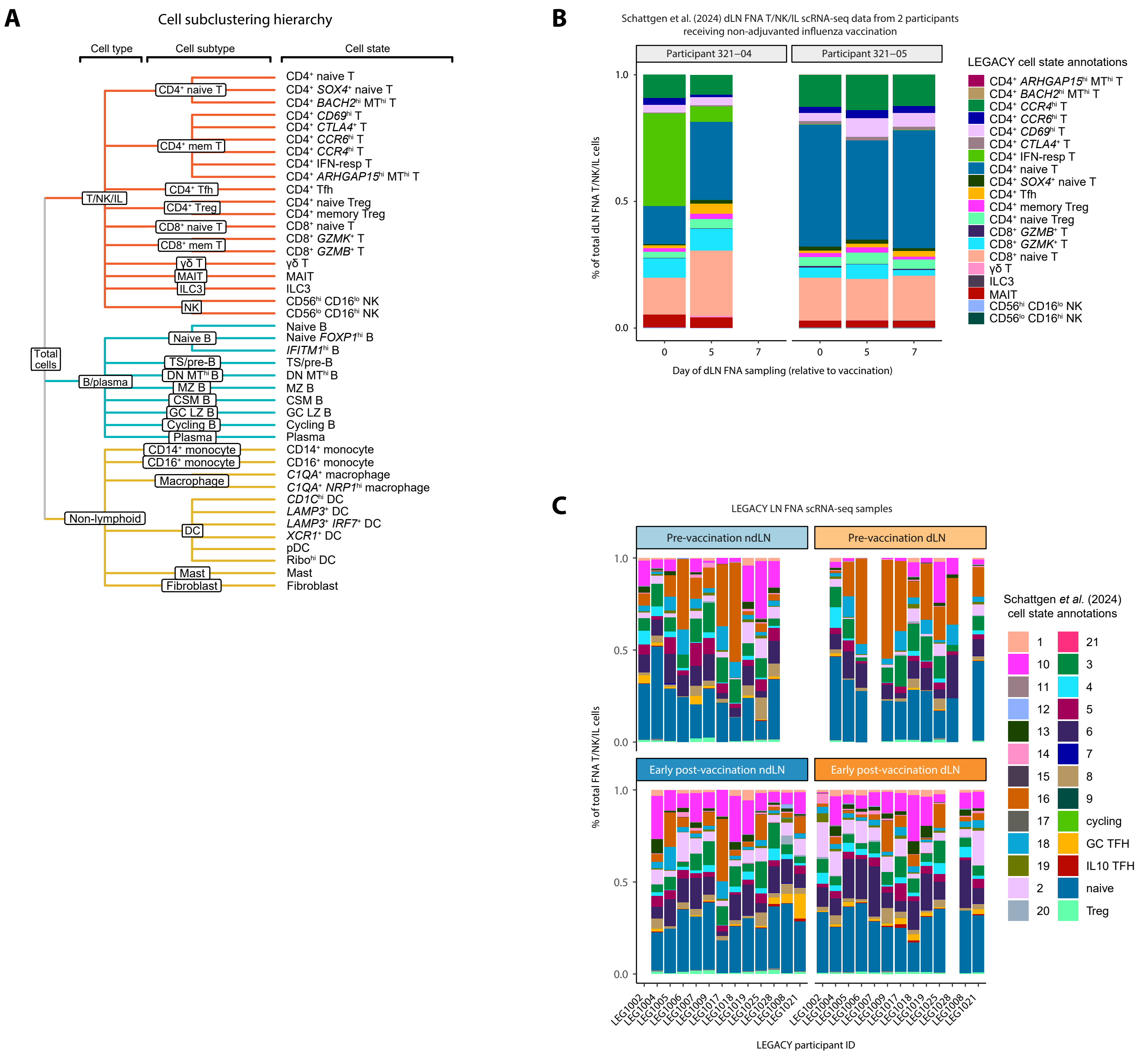


**Figure S6. LEGACY study cell subclustering hierarchy and LN annotation reference mapping across studies.** **(A)** LEGACY study LN FNA cell subclustering at three resolutions. **(B)** LEGACY study annotations mapped onto T/NK/IL scRNA-seq data from the dLN of two participants from the Schattgen et al.[17] study. Proportions of the different cell states are shown for each sampling time-point (before and early after vaccination with a non-adjuvanted influenza vaccine). **(C)** Mapping of the Schattgen et al.[17] cell cluster annotations onto the LEGACY data. Cell cluster proportions are shown for each cluster.

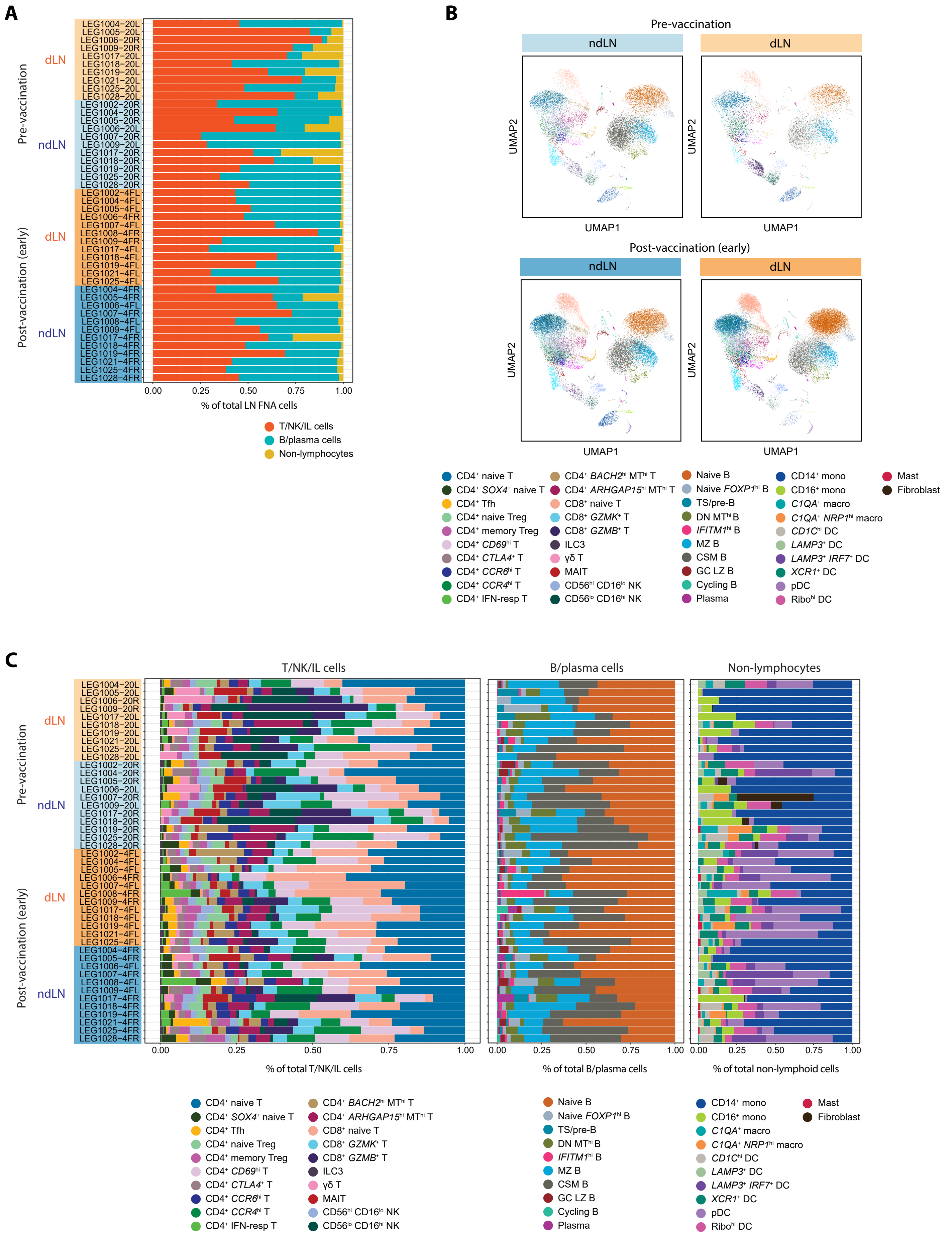


**Figure S7. LN FNA-derived scRNA-seq cell type and cell state distributions by sample.**
**(A)** Stacked barplot of per sample % of T/NK/IL, B/plasma and non-lymphoid cells out of the total cells. **(B)** UMAPs of all cell states faceted by LN site and vaccination time-point. **(C)** Stacked barplots of per sample % of cell states out of the total T/NK/IL, B/plasma or non-lymphoid cells.

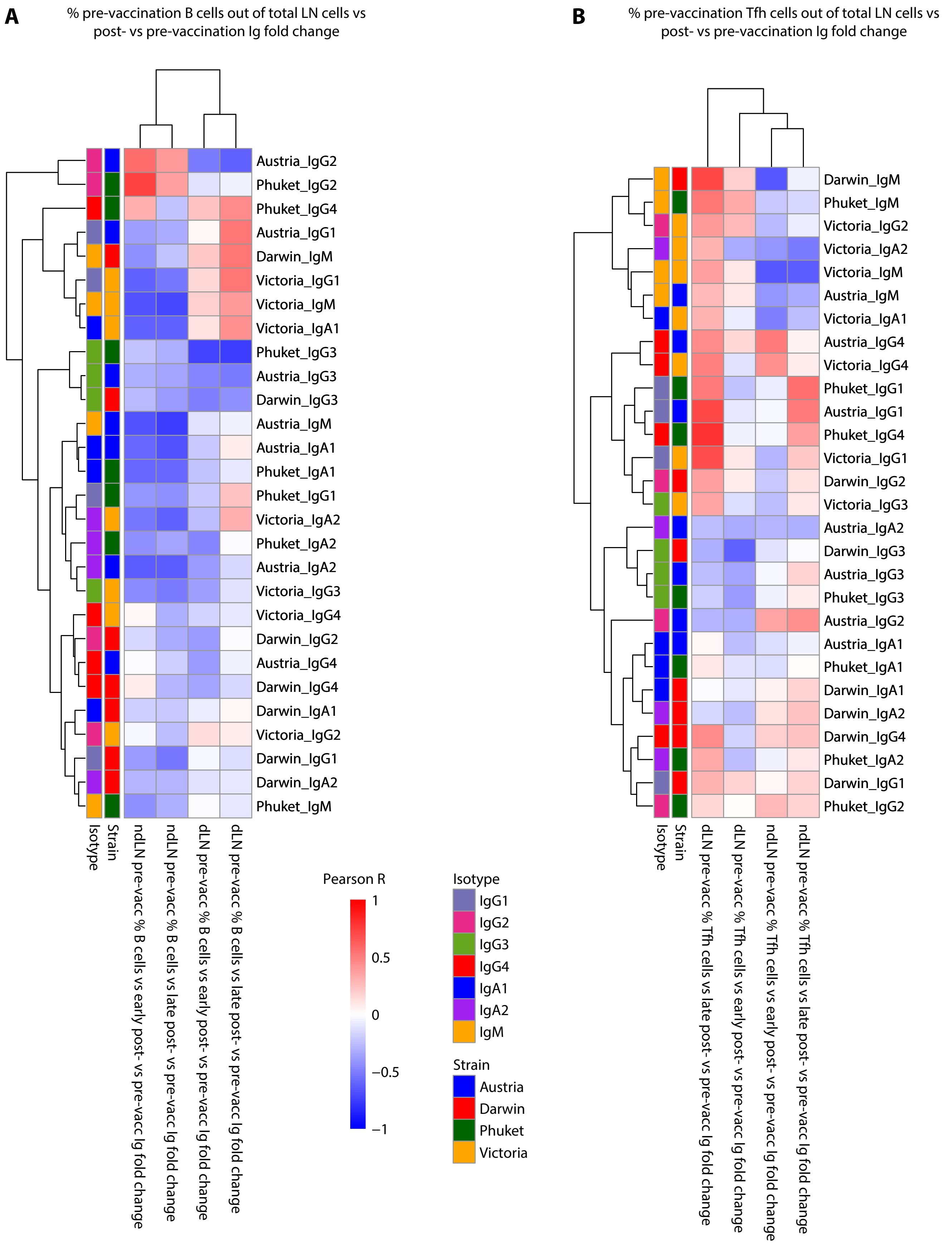


**Figure S8. Correlations between pre-vaccination % B and Tfh cells and the fold change in antigen-specific antibody titres.**

Pearson correlation of the % of **(A)** B cells or **(B)** Tfh cells out of the total dLN or ndLN cells pre-vaccination vs the fold change in antigen-specific Ig titres (detected by Luminex assay) at the early or late post- vs pre-vaccination time-points.

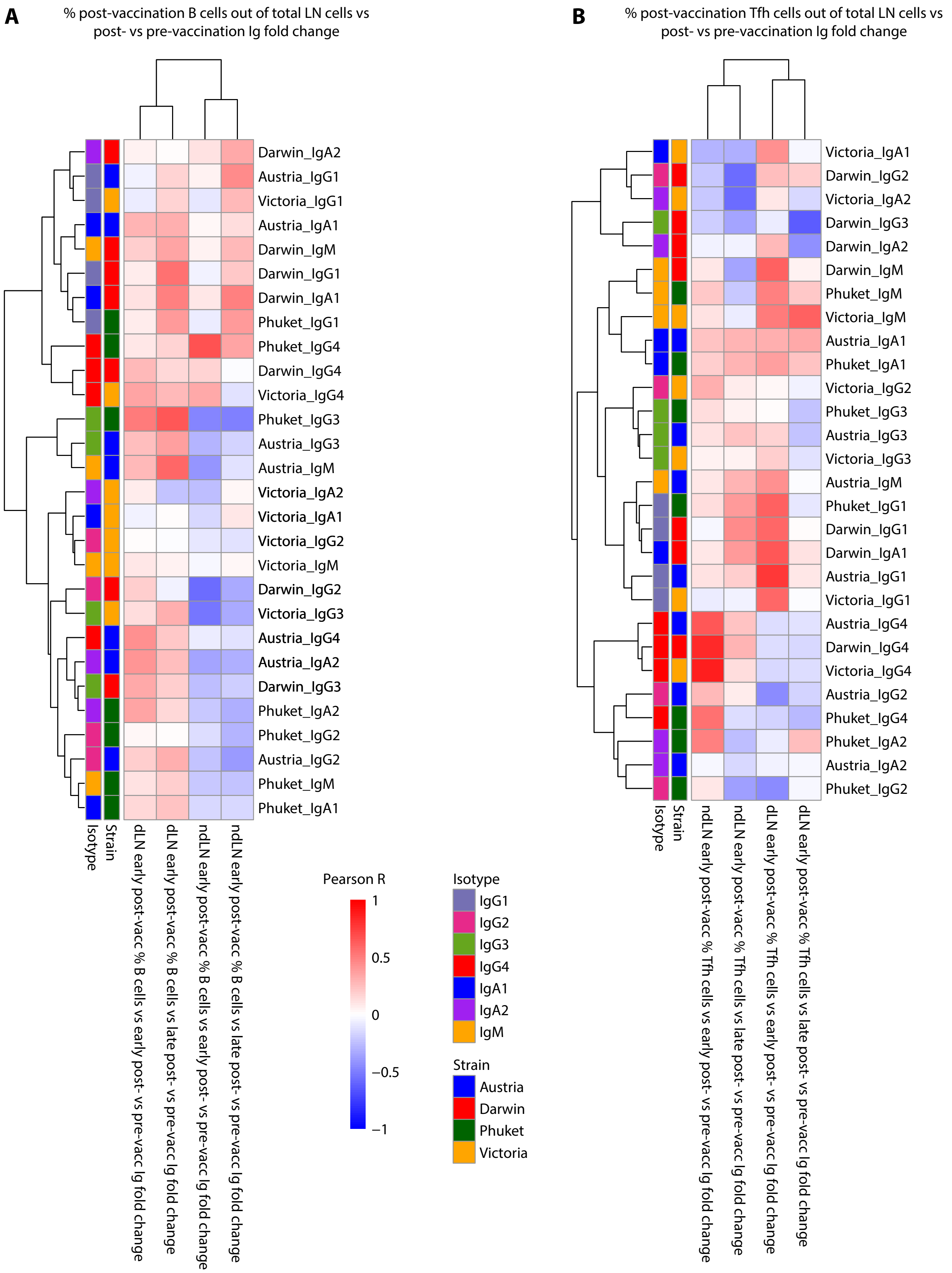


**Figure S9. Correlations between post-vaccination % B and Tfh cells and the fold change in antigen-specific antibody titres.**

Pearson correlation of the % of **(A)** B cells or **(B)** Tfh cells out of the total dLN or ndLN cells at the early post-vaccination (vacc) time-point vs the fold change in antigen-specific Ig titres (detected by Luminex assay) at the early or late post- vs pre-vaccination time-points.
