# Supplementary material for: Early lymph node T follicular helper cell signalling hub drives influenza vaccine response in an ancestrally diverse cohort
Source: eBioMedicine. 2025 Nov 29;122:106036. doi: 10.1016/j.ebiom.2025.106036 (PMC12703867; doi:10.1016/j.ebiom.2025.106036)
Supplement: Supplementary Material [file mmc6.pdf]

An experimental medicine study of seasonal influenza vaccination responses in Lymph nodE single-cell Genomics in AnCestrY (LEGACY01)

Version 3.1 06 Jul 2023

MAIN SPONSOR: Imperial College London

FUNDERS: Chan Zuckerberg Initiative

STUDY COORDINATION CENTRE: NIHR Imperial Clinical Research Facility

IRAS Project ID: 314444

Protocol authorised by:

Name	Role	Date	Signature
Katrina Pollock	Chief Investigator	06 Jul 2023	
Calliope Dendrou	Scientific Lead	06 Jul 2023	

The LEGACY Network is a collaboration between three partner institutions, Imperial College London, UK, the University of Oxford, UK and the Uganda Virus Research Institute, Uganda.

Imperial College London

UNIVERSITY OF OXFORD

© Imperial College of Science, Technology and Medicine

1. GENERAL INFORMATION

This protocol was constructed using the Imperial College Research Governance and Integrity Team template (Template Ref: RGIT_TEMP_027, Template V6.0 04Nov2021). The authors wish to credit the MRC CTU at UCL for use of their Protocol Template version 8.0 in drafting of this protocol, which describes the LEGACY01 study and provides information about procedures for entering participants. Every care was taken in its drafting, but corrections or amendments may be necessary. These will be circulated to investigators in the study. Problems relating to this study should be referred, in the first instance, to the Chief Investigator.

COMPLIANCE

The study will be conducted in compliance with the approved protocol, the Declaration of Helsinki 1996, the principles of Good Clinical Practice (GCP) ICH topic E6 (R2), and revision E6 (R3) EWG, General Data Protection Regulation and the UK Data Protection Act 2018, and the UK Policy Framework for Health and Social Care Research.

STUDY MANAGEMENT GROUP

Chief Investigator
Dr Katrina Pollock
NIHR Imperial Clinical Research Facility
ICTEM Building, Hammersmith Hospital Campus
Du Cane Road
London W12 0HS
UK
Tel: 020 3313 8070 Email: katrina.pollock@nhs.net, k.pollock@imperial.ac.uk

PROTOCOL DEVELOPMENT TEAM

Name	Address	Email and telephone
Mark Coles (MC)	Kennedy Institute of Rheumatology, University of Oxford Oxford, OX3 7FY	mark.coles2@kennedy.ox.ac.uk<br>(44) 1865 612675
Calli Dendrou (CD)	Wellcome Centre for Human Genetics, University of Oxford, Oxford OX3 7BN	cdendrou@well.ox.ac.uk<br>calliope.dendrou@imm.ox.ac.uk<br>(44) 1865 287 657
Pontiano Kaleebu (PK)	Uganda Virus Research Institute, Plot 51-57 Nakiwogo Road, PO Box 49, Entebbe, Uganda	pontiano.kaleebu@mrcuganda.org<br>Tel: Direct: +256 (0) 417 704103;<br>Tel: PA +256 (0) 417 704145<br>Cell phone +256-772 500 905

© Imperial College of Science, Technology and Medicine

Hashem Koohy (HK)	MRC Human Immunology Unit, MRC Weatherall Institute of Molecular Medicine University of Oxford Headley Way OX3 9DS	hashem.koohy@rdm.ox.ac.uk
Teresa Lambe (TL)	Oxford Vaccine Group University of Oxford, Oxford, OX3 7LE	teresa.lambe@paediatrics.ox.ac.uk
Brian Marsden (BM)	Kennedy Institute of Rheumatology NDORMS University of Oxford Old Road Campus Roosevelt Drive Headington OX3 7FY	brian.marsden@cmd.ox.ac.uk<br><br>(44) 1865 612658
Anita Milicic (AM)	The Jenner Institute<br><br>Old Road Campus Research Building, Roosevelt Drive, Oxford OX3 7DQ	anita.milicic@ndm.ox.ac.uk<br><br>(44) 1865 617613
Aime Palomeras (AP)	NIHR Imperial Clinical Research Facility, Hammersmith Hospital Du Cane Rd, London W12 0HS	aime.boakye@nhs.net<br><br>(44) 20 3313 8070
Samantha Vanderslott (SV)	Oxford Vaccine Group University of Oxford, Oxford, OX3 7LE	samantha.vanderslott@paediatrics.ox.ac.uk<br><br>(44) 1865 857420

Patient and public involvement and engagement (PPIE) representatives:

Two PPIE representatives have reviewed the study protocol prior to regulatory submission:

Dolapo Ogunleye, Patient Representative for NHS DigiTrials and PPIE representative for the LEGACY Network

Saira Tamboo PPIE representative for the LEGACY Network

Statisticians:

Calliope Dendrou, Sir Henry Dale Fellow, Nuffield Department of Medicine, University of Oxford

Hashem Koohy, Associate Professor of Systems Immunology, University of Oxford

© Imperial College of Science, Technology and Medicine

STUDY SITE AND COORDINATION CENTRE

NIHR Imperial Clinical Research Facility (Imperial CRF)

Hammersmith Hospital

Imperial College Healthcare NHS Trust

Du Cane Road

London W12 0HS

For general queries, supply of study documentation, and collection of data, please contact:

Study Coordinator: Aime Palomeras

Tel: 020 3313 8070 E-mail: aime.boakye@nhs.net

Web address: https://www.imperial.ac.uk/nihr-crf/

CLINICAL QUERIES

Clinical queries should be directed to Katrina Pollock who will direct the query to the appropriate person.

SPONSOR

Imperial College London is the main research Sponsor for this study. For further information regarding the sponsorship conditions, please contact the Head of Regulatory Compliance at:

Research Governance and Integrity Team
Imperial College London and Imperial College Healthcare NHS Trust
Room 215, Level 2, Medical School Building
Norfolk Place
London, W2 1PG
Tel: 0207 594 1862
Imperial College - Research Governance and Integrity Team (RGIT) Website

FUNDER

Chan Zuckerberg Initiative

STUDY REGISTRATION

The LEGACY01 study is registered with the ISRCTN registry, ISRCTN13657999.

© Imperial College of Science, Technology and Medicine

2. GLOSSARY OF ABBREVIATIONS

Abbreviation	Expansion
BMI	Body mass index
COVID-19	Coronavirus disease 19
CRF	Clinical Research Facility
CRN	Clinical Research Network
DNA	Deoxyribonucleic acid
FDA	(US) Food and Drug Administration
GCP	Good Clinical Practice
GP	General Practitioner
HCA	Human Cell Atlas
HIV	Human Immunodeficiency Virus
HRA	Health Research Authority
ICRF	Imperial Clinical Research Facility
IM	Intramuscular
IRAS	Integrated Research Application System
LNC	Lymph node cells
MHRA	Medicines and Healthcare products Regulatory Agency
mL	Millilitre
MRC	Medical Research Council
RNA	Ribonucleic acid
NHS	National Health Service
NIHR	National Institute for Health Research
NRES	National Research Ethics Service
PBMC	Peripheral blood mononuclear cells
PI	Principal Investigator

© Imperial College of Science, Technology and Medicine

PPIE	Patient and public involvement and engagement
QC	Quality control
QIV	Quadrivalent influenza vaccine
REC	Research Ethics Committee
RNA	Ribonucleic acid
SAE	Serious adverse event
SmPC	Summary of Product Characteristics
Ultrasound	US
WHO	World Health Organization

KEYWORDS

Single-cell sequencing; lymph node; influenza vaccine; ancestry

© Imperial College of Science, Technology and Medicine

3. STUDY SUMMARY

TITLE	An experimental medicine study of seasonal influenza vaccination responses in Lymph nodE single-cell Genomics in AnCestrY
ACRONYM	LEGACY01
IRAS ID	314444
SPONSOR	Imperial College London
DESIGN	Experimental medicine study; single arm, non-randomised, open label
SETTING	Secondary care (NHS) and academic research facilities
AIM	To investigate human immune responses in lymph node cells before and after immunisation with a seasonal influenza vaccine
OBJECTIVES	Primary objective: To generate a single cell atlas of lymph node cells before and after immunisation with seasonal influenza vaccine.<br>Secondary objective: To compare serum antibody responses before and after immunisation.<br>Exploratory: To compare cellular immune responses in various immune compartments e.g. blood and lymph nodes, against antigens including influenza before and after immunisation with seasonal influenza vaccine.<br>Exploratory: To compare immune responses in various immune compartments (e.g., blood and lymph nodes) against antigens including influenza before and after immunisation to help inform vaccine development and testing across different ethnicities.<br>Capacity building and training: To build capacity with respect to staff expertise and resource between the three partner institutions to support this project and future similar research.
OUTCOME MEASURES	Outcome measures may include but are not limited to the following assays<br>1. Single cell RNA sequencing analysis of LNC and matched paired PBMC<br>2. Binding ELISA specific for influenza/A antigens e.g. haemagglutinin<br>3. Intracellular cytokine secretion or activation induced marker assay of PBMC and LNC<br>4. Genotypic assays of areas of the genome of immunological relevance may include tests such as HLA-testing.
POPULATION	Healthy adults aged 18 – 55 years n=30<br>Cohort 1 in influenza season 2022 to 2023<br>Cohort 2 in influenza season 2023 to 2024

© Imperial College of Science, Technology and Medicine

ELIGIBILITY	Individuals with African or Asian ancestry
DURATION	Three years

© Imperial College of Science, Technology and Medicine

4. FLOW DIAGRAM AND STUDY SCHEDULE

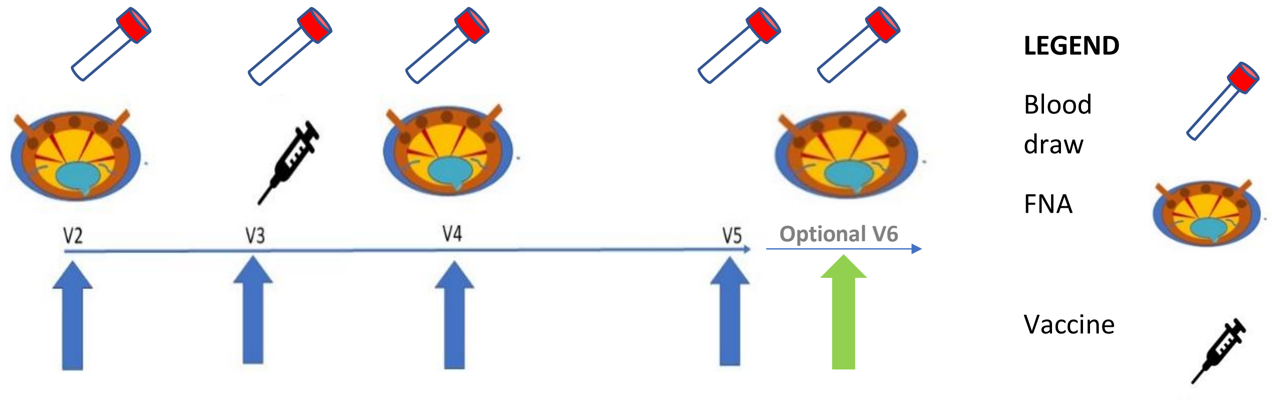

Figure 3. Timeline of study sampling and vaccination events for enrolled participants including lymph node FNA and influenza vaccination. Participants enrol at Visit 2 and undergo paired peripheral blood and lymph node sampling, vaccination at Visit 3, repeat paired lymph node sampling at Visit 4 and then phlebotomy at Visit 5. Additional paired lymph node sampling at Visit 6 offered to cohort 2 participants as an optional visit.

.

© Imperial College of Science, Technology and Medicine

Table 1. Schedule of investigations, treatments, and assessments

Study visit	V1	V2	V2a	V3	V4	V5	V6	V6a
Visit location	Site	Site	Remote (by phone)	Site	Site	Site	Site	Remote (by phone)
Visit type	Screening	Enrolment: FNA 1	Follow up	Vaccination	Follow-up: FNA2	Follow up	Optional follow up FNA 3[5]	Follow up
Study week	minus 24 to minus 1	0	0	1	2	5	7	7
Study day[4]	minus 168 to minus 1	0	5	7	12	35	49	54
Window (days)	NA	NA	minus 1 to plus 1	0 to plus 161	minus 2 to plus 2*	minus 2 to plus 14	minus 2 to plus 28	minus 1 to plus 1
Informed consent	x							
Demographics	x							
Medical history	x							
Weight and height, calculate BMI	x							
Blood borne virus screen (approx. 6mL)[1]	x							
Laboratory safety tests (approx. 10mL)[2]	x							
Concomitant medication[3]	x	x	x	x	x	x	x	x
COVID-19 symptoms and trigger COVID-19 test[3]	x	x	-	x	x	x	x	
Urinary pregnancy test[3]	x			x				
Symptom directed physical examination[3]	x	x		x	x	x	x	
Inspection of the FNA site[3]	x	x		x	x	x	x	
Vital signs[3]	x	x		x	x	x	x	
Ultrasound scan[3]		x			x		x	
Lymph node fine needle aspiration		x			x		x	
Vaccination				x				
Blood for serum immunoassays (6mL)[3]		x		x	x	x	x	
Blood for cellular and plasma immunoassays (42mL)[3]		x		x	x	x	x	

© Imperial College of Science, Technology and Medicine

Blood for RNA PAXgene tube (2.5mL)[3]				x	x			
Blood for HLA testing (3-4 mL)[3]		x						
Adverse events check[3]		x	x	x	x	x	x	x
Participant feedback survey[6]						x		
Blood volume (approx.) (mL)	16	52	-	50.5	50.5	48	48	-

1. Detection of antibodies and/or antigen for HIV, hepatitis B and hepatitis C
2. Full blood count, liver function, renal function, non-fasting glucose
3. At visits which include FNA or vaccination, there will be an AE check and vital signs pre-FNA/pre-vaccination, and again at least 30 min after. At visits which include FNA, there will be an inspection of the FNA site pre-FNA, and again at least 30 min after. All other procedures/assessments at these visits will be pre-FNA/pre-vaccination only.
4. The timings of V2a and V3 are set according to that of V2; the timings of V4, V5 and V6 are set according to that of V3.
5. Optional third FNA for up to n=6 cohort 2 participants only at 6 weeks post vaccination followed by a final phone visit to discuss any AEs.
6. A paper survey at visit 5 for cohort 2 only to collect information on participant's experience.

* This is the preferred window. However, if the FNA2 visit cannot be scheduled within the preferred window, it can take place up to 21 days after the vaccination without being a protocol deviation. Every effort should be made to schedule the FNA2 visit as close to 5 days post-vaccination as possible.

© Imperial College of Science, Technology and Medicine

5. TABLE OF CONTENTS

1. GENERAL INFORMATION 2
2. GLOSSARY OF ABBREVIATIONS 5
3. STUDY SUMMARY 7
4. FLOW DIAGRAM AND STUDY SCHEDULE 9
5. TABLE OF CONTENTS 12
6. INTRODUCTION 13
7. STUDY DESIGN 14
8. PARTICIPANT ENTRY 16
9. ADVERSE EVENTS 18
10. ASSESSMENT AND FOLLOW-UP 19
11. QUALITY ASSURANCE AND CONTROL 22
12. STATISTICS AND DATA ANALYSIS 22
13. REGULATORY ISSUES 24
14. INDEMNITY 25
15. FUNDING 25
16. STUDY MANAGEMENT 25
17. PATIENT AND PUBLIC INVOLVEMENT 26
18. PUBLICATION POLICY 26
19. DATA AND SAMPLE SHARING 26
20. PROTOCOL AMENDMENTS 27
21. REFERENCES 28
22. APPENDIX 1. INFLUENZA VACCINE USE IN THE UK 30
23. APPENDIX 2. FINE NEEDLE ASPIRATION OF THE LYMPH NODE 35
24. APPENDIX 3. POST MARKETING SURVEILLANCE FOR FLUAD 36

© Imperial College of Science, Technology and Medicine

6. INTRODUCTION

BACKGROUND

The efficient development of vaccines effective against global and emerging pathogens is complicated by geographic differences in vaccine responsiveness. The relative contribution of genetic and environmental factors to differential immune responses after vaccination remains poorly defined and is complicated by the spectrum of immune cell types involved. The Lymph nodE single-cell Genomics AnCestrY LEGACY01 study will create an ethnically diverse dataset with a single-cell atlas of lymph node (LN) and blood vaccine responses. Healthy volunteers of African and Asian ancestry who have immigrated to or were born in the UK will be invited to participate. They will be asked to donate blood and lymph node samples at specific timepoints pre- and post-immunisation. LN fine-needle aspirates (FNAs) will focus on the dynamics of germinal centre formation, capturing the interplay between lymphocytes, antigen-presenting cells and the stromal microenvironment. This will generate a curated data-structure of FNA single-cell (sc)RNA-seq, blood transcriptomic, cytometric, and serological analyses, with ethnicity, genetic, demographic and clinical metadata. To facilitate atlas utilisation and enable future international adoption of this approach, data, protocols and training materials will be made open access.

RATIONALE FOR CURRENT STUDY

Recent infectious disease outbreaks, including the COVID-19 pandemic, have emphasized the critical need for rapid development of effective vaccines.[1-5] However, recognised variation in immune responses after vaccination across populations in different countries poses a barrier to progress, and it has yet to be determined whether this variation is due to differences in genetic background, in environmental influences including past or persistent antigenic exposure, and/or other poorly defined mechanisms.[6-9] Despite this known variation, the majority of mechanistic studies investigating immune responsiveness to vaccines have focused on populations of white European ancestry.[10,11] In the UK, this underrepresentation of ethnic minorities in vaccine experimental medicine research is at least partly due to vaccine hesitancy amongst these groups.[12]

A further challenge in addressing differences in vaccine response across populations is that studies have traditionally focused on the use of peripheral blood samples, even though inflammatory and germinal centre (GC) responses mounted in the draining LNs likely dictate vaccine priming efficiency and underpin protective efficacy.[13] Ultrasound (US) guided FNA is a tractable and safe approach for LN sampling, and its use has recently demonstrated a good representation of cell subtypes found in excisional biopsies, including GC cells, thereby opening invaluable new routes for assessing vaccine efficacy in humans in vivo.[14-17]

The LEGACY01 clinical study will address this by creating a dataset of unstimulated and stimulated in vivo immune responses to a model, seasonal influenza vaccine, from participants with African and Asian ancestry.

ROLE OF THE PARTNER INSTITUTIONS

Imperial College London is the Sponsor for the study. Recruitment and study visits will be at the NIHR Imperial Clinical Research Facility, Hammersmith Hospital, Du Cane Road, London W12 0HS.

© Imperial College of Science, Technology and Medicine

Imperial College London and Imperial CRF will manage the study. The University of Oxford will receive and process samples and run laboratory analysis of blood and lymph node tissues led by Dr Calli Dendrou at the Wellcome Centre for Human Genetics. The Uganda Virus Research Institute will support establishment of a framework for delivering single-cell vaccine studies at international sites such as Uganda. Specifically, they will appoint and support the training of a visiting Clinical Research Fellow to the UK at Imperial College London and the University of Oxford. All centres will support the patient and public involvement and engagement activities of the study.

HUMAN CELL ATLAS

LEGACY01 is one of the Ancestry Networks for the Human Cell Atlas funded by the Chan Zuckerberg Initiative. The goal of these networks is to generate single-cell data from ancestrally diverse tissue samples, which will be uploaded and made available as open access research data for the Human Cell Atlas[18].

STUDY AIM, OBJECTIVES AND OUTCOME MEASURES

The aim of the study is to investigate human immune responses in lymph node cells before and after immunisation with a seasonal influenza vaccine

Primary objective: To generate a single cell atlas of lymph node cells before and after immunisation with seasonal influenza vaccine.

Secondary objective: To compare serum antibody responses before and after immunisation.

Exploratory: To compare immune responses in various immune compartments (e.g., blood and lymph nodes) against antigens including influenza before and after immunisation to help inform vaccine development and testing across different ethnicities.

Capacity building and training: To build capacity with respect to staff expertise and resource between the three partner institutions to support this project and future similar research.

Outcome measures may include but are not limited to the following assays:

1. Single cell RNA sequencing analysis of LNC and matched paired PBMC
2. Binding ELISA specific for influenza/A antigens e.g., haemagglutinin
3. Intracellular cytokine secretion or activation induced marker assay of PBMC and LNC
4. Genotypic assays such as HLA-testing.

7. STUDY DESIGN

Interventional experimental medicine study using a model vaccine, seasonal influenza vaccine, as a probe to stimulate the immune system in vivo with sampling to collect serum, plasma, PBMC and LNC at specified time points pre and post immunisation. The study is single arm, non-randomised and open label.

Duration: Three years

© Imperial College of Science, Technology and Medicine

Number and type of subjects: Thirty healthy adult volunteers of African or Asian ancestry will be enrolled. Participants may enrol into either cohort 1 (influenza season 2022-2023) or cohort 2 (influenza season 2023-2024).

Study intervention: A single dose of a licensed seasonal influenza vaccine will be administered intramuscularly into the deltoid muscle. See Appendix 1 for (2021-2024) recommendations for seasonal influenza vaccines in the UK.

INFLUENZA VACCINE

The influenza vaccine of choice for this study is adjuvanted quadrivalent influenza vaccine (aQIV), also marketed as Fluad Tetra (Seqirus UK Ltd). One 0.5 mL dose of aQIV contains 15 micrograms of haemagglutinin from two A and two B strains of influenza propagated in hens' eggs. It is adjuvanted with MF59C.1 which contains per 0.5ml dose, squalene (9.75 mg), polysorbate 80 (1.175 mg), sorbitan trioleate (1.175 mg), sodium citrate (0.66 mg) and citric acid (0.04 mg).

Clinical studies have been conducted in individuals aged 65 years and over and in children aged 6 months to 6 years. In studies V118_20 and V118_18, n=4269 subjects aged 65 years and older were vaccinated. AEs were injection site pain (16.3% and 31.9%), fatigue (10.5% and 16.0%) and headache (10.8% and 12.0%) and resolved within three days. In the first study, during the 2017-2018 Northern Hemisphere influenza season Fluad Tetra met non-inferiority for influenza A/H1N1, A/H3N2, B/Yamagata and B/Victoria and superiority for the B strains not included in the Fluad aTIV comparators, aTIV-1 and aTIV-2[19,20]

Study V118_05 assessed Fluad Tetra in children aged 6 months to 6 years and elicited a higher immune response than the non-adjuvanted vaccine that persisted to 12 months. A higher incidence of local and systemic reactions was reported. Although acceptable immunogenicity was reported, the vaccine did not meet its primary efficacy endpoint in this paediatric study.

Safety and immunogenicity data of Fluad, the trivalent formulation, is relevant for aQIV because they have similar compositions and are manufactured in the same way. Post marketing surveillance data are not available yet for aQIV but are available for Fluad and are detailed in Appendix 3. [21]

The aQIV is licensed for clinical use in patients aged 65 years and over due to its immunogenicity profile in this age group. Coupled with the paediatric data, it is expected that this vaccine will deliver high titres of influenza-specific antibody at least equivalent to the unadjuvanted vaccine which includes 15 µg of haemagluttinin per strain and which is currently widely used in younger adults. aQIV contains haemagglutinin from four strains of influenza virus. As a result, participants choosing to enter this study during the Northern hemisphere influenza season and who receive the study vaccine are not expected to be disadvantaged compared with individuals who receive an influenza vaccine through the NHS.

An adjuvanted vaccine is chosen for this experimental medicine study in order to investigate lymph node responses to immunisation where the adjuvant will stimulate a local innate response. It is expected that swelling of the cortex will be visible using greyscale (or conventional) US. The published safety and tolerability profile of aQIV is in line with expected responses for an influenza vaccine. A similar safety and tolerability profile is expected in adults aged 18 – 55 years.

© Imperial College of Science, Technology and Medicine

Participants will undergo a fine needle aspiration of a suitable axillary lymph node on two occasions with an optional third visit for up to n=6 cohort 2 only at sites ipsilateral and contralateral to the site of injection.

8. PARTICIPANT ENTRY

PRE-REGISTRATION EVALUATIONS

Participants will attend a screening visit and provide written informed consent to enrol onto the study.

PARTICIPANT INCLUSION CRITERIA

- Healthy adults aged 18 years to 55 years on the day of screening
- Willing and able to provide written informed consent
- Identifies as having African or Asian ancestry[i]
- Usually resident in the UK for at least 5 years prior to screening
- If female and of childbearing potential[ii] not pregnant on the day of screening and willing to use a highly effective form of contraception until 12 weeks after the study immunisation[iii].
- Willing to avoid all other vaccines within 4 weeks either side of the study injection and fine needle aspiration[iv].
- Willing and able to comply with the visit schedule and provide samples.
- Willing to grant authorised persons access to his/her trial related medical record and GP records either directly or indirectly.

i A list of ethnic groups in the UK is available at [List of ethnic groups - GOV.UK (ethnicity-facts-figures.service.gov.uk)].

ii A woman will be considered of childbearing potential following menarche and until becoming post-menopausal unless permanently sterile. Permanent sterilisation methods include hysterectomy, bilateral salpingectomy and bilateral oophorectomy. A post-menopausal state is defined as no menses for 12 months without an alternative medical cause.

iii Highly effective forms of contraception include medical sterilisation of a women or her partner, hormonal methods of contraception that prevent ovulation and intrauterine devices or systems. Barrier methods of contraception are not considered highly effective. Abstinence should be the preferred and usual lifestyle of the participant.

iv An exception is made for authorised or licensed COVID-19 vaccination according to national clinical directives. A gap of at least 28 days either side of an FNA is preferred but where there is clinical imperative a shorter gap is acceptable. Where a COVID-19 vaccine is scheduled near to an FNA, the FNA should occur before the vaccination where possible.

PARTICIPANT EXCLUSION CRITERIA

- Pregnant or lactating
- Has a significant clinical history, physical finding on clinical examination or laboratory finding during screening, or presence of a disease that is active or requires treatment to control it,

© Imperial College of Science, Technology and Medicine

including cardiac, respiratory, endocrine, metabolic, autoimmune, liver, neurological, oncological, psychiatric, immunosuppressive/immunodeficient or other disorders which in the opinion of the investigator is not compatible with healthy status, may compromise the volunteer's safety, preclude vaccination or tissue sampling or compromise interpretation of the immune response to vaccine. Individuals with mild/moderate, well-controlled comorbidities are allowed.

- Body mass index of 35 or greater
- History of anaphylaxis or angioedema
- History of severe or multiple allergies to drugs or pharmaceutical agents or contraindicated from receiving influenza vaccine or local anaesthetic including lidocaine.
- History of severe local or general reaction to vaccination defined as:

local: extensive, indurated redness and swelling involving most of the arm, not resolving within 72 hours

general: fever ≥39.5 °C within 48 hours; bronchospasm; laryngeal oedema; collapse; convulsions or encephalopathy within 72 hours

- Receipt of any immunosuppressive agents within 18 weeks of screening by any route other than topical
- Prescribed regular blood thinning medication likely to induce bruising or bleeding on fine needle aspiration
- Detection of antibodies to hepatitis C
- Detection of antibodies to HIV
- Detection of anti-hepatitis B core antibodies
- Participating in a clinical trial with an investigational drug or device or treated with an investigational drug within 28 days of screening.

WITHDRAWAL CRITERIA

Participants are free to withdraw from the study at any time and for any reason without prejudicing their usual medical care.

Discontinuing sample donation: those participants who choose to discontinue lymph node tissue donation can be followed up to donate blood samples. Those participants where the investigator chooses to discontinue tissue donation can be followed up to donate blood samples.

Participants who choose to discontinue all (blood and lymph node) sample donation will be withdrawn from the study once the investigator deems it safe to do so.

Withdrawing from vaccination: Those participants who choose not to, or are unable to, have an influenza vaccine as part of the study schedule can continue to provide lymph node and blood samples.

Additional volunteers may be enrolled to replace withdrawn participants or those who are unable to complete the study schedule of visits per protocol for any reason.

© Imperial College of Science, Technology and Medicine

COENROLMENT GUIDELINES

Participants will be entered into the TOPS database to prevent over-volunteering. Co-enrolment into another study is discouraged but can be allowed at the discretion of the investigator where neither the safety of the participant nor the integrity of the data is at risk. An example would be co-enrolment into an observational study with either no sampling or a low volume blood draw.

9. ADVERSE EVENTS

DEFINITIONS

Adverse Event (AE): any untoward medical occurrence in a patient or clinical study subject.

Serious Adverse Event (SAE): any untoward medical occurrence or effect that:

Results in death

Is life-threatening – refers to an event in which the subject was at risk of death at the time of the event; it does not refer to an event which hypothetically might have caused death if it were more severe

Requires hospitalisation, or prolongation of existing inpatients' hospitalisation

Results in persistent or significant disability or incapacity

Is a congenital anomaly or birth defect

Medical judgement should be exercised in deciding whether an AE is serious in other situations. Important AEs that are not immediately life-threatening or do not result in death or hospitalisation but may jeopardise the subject or may require intervention to prevent one of the other outcomes listed in the definition above, should also be considered serious.

REPORTING PROCEDURES

Adverse events should be reported depending on the nature of the event and the reporting procedures below should be followed. Any questions concerning adverse event reporting should be directed to the Chief Investigator in the first instance. Events should be graded according to the FDA: Toxicity Grading Scale for Healthy Adult and Adolescent Volunteers Enrolled in Preventive Vaccine Clinical Trials, taking account of local laboratory reference ranges. https://www.fda.gov/media/73679/download

NON-SERIOUS AES

These will be recorded in the following instances:

Those occurring in the 5 days following lymph node tissue sampling

Those occurring in the 28 days following influenza vaccination

© Imperial College of Science, Technology and Medicine

SERIOUS AES

SAEs must be recorded during the entire study.

An SAE form should be completed and emailed to the Chief Investigator within 24 hours. However, hospitalisations for elective treatment of a pre-existing condition do not need reporting as SAEs.

All SAEs should be reported to the REC which gave favourable opinion of the study, where in the opinion of the Chief Investigator the event was:

'related', i.e. resulted from the administration of any of the research procedures; and

'unexpected', i.e. an event that is not listed in the protocol as an expected occurrence

Reports of related and unexpected SAEs should be submitted within 15 days of the Chief Investigator becoming aware of the event, using the NRES SAE form for non-IMP studies. The Chief Investigator must also notify the Sponsor of all related and unexpected SAEs.

The aQIV is subject to additional monitoring. At the Chief Investigator's discretion, side effects of vaccination that are suspected adverse reactions will be reported to the MHRA via its Yellow Card Scheme: www.mhra.gov/yellowcard

CONTACT DETAILS FOR REPORTING SAES
Please send SAE forms to the Chief Investigator: katrina.pollock@nhs.net
Tel: +44(0)203 313 8073 (Mon to Fri 09.00 – 17.00)
The Chief Investigator is responsible for onward reporting of SAEs to the REC and Sponsor (RGIT@imperial.ac.uk)

10. ASSESSMENT AND FOLLOW-UP

Participants who may be eligible will be identified through access to the NIHR Imperial CRF healthy volunteer database, and through advertising the study across multiple outlets including in the community, in the workplace and online. Written information about the study will be made available on request. Interested potential participants will be invited for a face-to-face screening visit.

STUDY ASSESSMENT SCHEDULE

Participants will be followed up according to the study schedule in Table 1. Participants will attend a screening visit at the study site, and they will be asked to provide proof of identity at the visit. Written informed consent will be obtained from participants before undergoing study procedures. The visits in the study assessment schedule are a screening visit (V1), an enrolment visit at which the first FNA will be performed (V2), a vaccination visit (V3), a follow-up visit at which the second FNA will be performed (V4) and a final follow-up visit (V5). In addition, participants will be contacted by telephone (V2a) a few days after the first FNA at V2. For a small number of participants in cohort 2 (up to n=6) there will be an option of a third FNA visit at 6 weeks post vaccination.

© Imperial College of Science, Technology and Medicine

PROCEDURES DURING THE SCREENING PERIOD

Informed consent

Participants will be given written information about the study, the sampling procedures, the vaccine and the data collection and sharing. Those who wish to proceed will be asked to sign a witnessed consent form before undergoing screening investigations. A copy will be provided to the participant, and one kept in the study file according to local SOPs.

Eligibility

Eligibility will be assessed by means of demographics, medical history, medications, physical examination and laboratory investigations. Screening examination will include clinical observations (blood pressure, temperature and pulse), height and weight, inspection of the FNA sites (skin and an assessment of lymph nodes). Symptom directed examination will be at discretion of the physician. During the study, participants should not take any aspirin or medication that may increase risk of bleeding 7 days before each procedure. Enrolment of participants who usually take these medications for any reason will be at the discretion of the investigator.

Clinical laboratory investigations

Tests to be conducted at screening are in Table 1 and its footnotes. Individuals with laboratory abnormalities at screening considered clinically significant by the investigator and likely to interfere with the conduct of the study will not be eligible for enrolment.

PROCEDURES AT ENROLMENT AND FOLLOW UP

Fine needle aspiration

Participants will undergo fine needle aspiration of suitable axillary lymph nodes at enrolment, and again after receiving immunisation. The process for sampling is described in Appendix 2.

Injection

Intramuscular injection of seasonal influenza vaccine into the deltoid muscle of participants' choice will be according to standard clinical practice. The arm injected will be recorded. Prior to receiving the injection, the participant will have vital signs measured. A temperature of 37.8 °C or above would prevent injection on the day. Introduction of new concomitant medications such as immunosuppressive medications, or new acute illness may prevent injection on the day at discretion of the investigator. Participants will remain at the study site for at least 30 min after injection, for safety reasons.

COVID-19

Participants will be asked about COVID-19 symptoms at every visit. Presence of highly likely COVID-19 symptoms in the opinion of the investigator will trigger a PCR or lateral flow test (which can be self-taken) in accordance with NHS guidance. The visit will be delayed until the result is known. A negative response will allow the visit to continue per protocol.

© Imperial College of Science, Technology and Medicine

A positive response will delay the visit until the NHS stipulated period of isolation is complete.

Receiving an authorised COVID-19 vaccine

It is anticipated that volunteers entering the study will already have received a full course of COVID-19 vaccination as defined by the Green Book chapter 14a. If they have not done so, volunteers are encouraged to receive a full course of COVID-19 vaccination at least 28 days before enrolling onto the study. Volunteers who enrol and subsequently become eligible for an authorised COVID-19 vaccine booster through standard NHS care can receive the vaccine during the study. A gap of at least 28 days either side of an FNA is preferred but where there is clinical imperative a shorter gap is acceptable. Where a COVID-19 vaccine is scheduled near to an FNA, the FNA should occur before the vaccination where possible.

PROCEDURES FOR ASSESSING SAFETY

Seasonal influenza vaccine is a widely used licensed product with a well described post-licensure safety profile. Detailed information on safety data available for aQIV is available in section 2 study design and in Appendix 1. aQIV is subject to additional monitoring and healthcare professionals are asked to report any suspected adverse reactions via the Yellow Card Scheme: www.mhra.gov.uk/yellowcard

Fine needle aspiration is a standard and well-tolerated clinical technique. Safety assessments on the study will be limited to assessing the safety and tolerability of study procedures purely in order to safeguard the wellbeing of participants, in accordance with the experimental medicine framework for the study. There are no safety-related objectives of the study.

PROCEDURES FOR ASSESSING IMMUNE RESPONSES

Whole blood and lymph node samples will be collected to assess cellular immunity and humoral immunity and DNA extracted for genomic analysis such as HLA typing. Whole blood will be collected at Visits 2 and 3-5. Lymph node samples will be collected at Visits 2 and 4. Pax gene tubes for assessing the RNA transcriptome will be collected at Visits 3 and 4.

INCIDENTAL FINDINGS

An incidental finding is one that has potential health or reproductive importance, which is discovered unexpectedly but is unrelated to the purpose or aims of the study e.g., an abnormal laboratory safety test result. Local site (NIHR Imperial CRF) procedures will be followed. Depending on the nature of the finding, the subject might have to be withdrawn or vaccination or sampling discontinued, and his/her GP informed.

LOSS TO FOLLOW-UP

Participants who do not attend scheduled visits will be contacted according to local standard procedures which includes contacting through telephone and NHS text. Every reasonable attempt will be made to maintain contact with participants during their participation in the study.

© Imperial College of Science, Technology and Medicine

STUDY CLOSURE

The study will be closed when all participants have completed their final follow-up visit and assessments are completed including those to determine resolution of any adverse events and the data recorded and locked.

11. QUALITY ASSURANCE AND CONTROL

Quality assurance (QA) includes all the planned and systematic actions established to ensure the study is performed and data generated, recorded and reported in compliance with the principles of GCP and any regulatory requirements. Quality control (QC) includes the operations to ensure that requirements for the study activities are fulfilled.

SAFETY AND RIGHTS OF PARTICIPANTS

Participants will be donating tissue and undergoing immunisation using procedures that are commonly undertaken for patients in the NHS. Confidential information will be kept allowing safety assessments to be made throughout the study.

PROJECT DESIGN AND RELIABILITY

A feasibility study is published, led by the Chief Investigator (EAVI2020_01 FNA sub study). This study has indicated that lymph node sampling is feasible, safe and with ample yields to be undertaken as part of human vaccine research[22].

CLINICAL DATA RECORDING AND MONITORING

Worksheets for the study will be created for each study visit. Completed worksheets will undergo regular QC assessments by the study clinical team. Data will be stored by means of an electronic database using REDCap or similar software system.

AUDITS

The study may be subject to audit by Imperial College London, under its remit as sponsor, and other regulatory bodies to ensure adherence to GCP and the UK Policy Framework for Health and Social Care Research.

12. STATISTICS AND DATA ANALYSIS

Thirty volunteers will be enrolled.

Ultrasound images will be collected using the software provided with the US machine operating system. These may be securely shared for storage on a secure password protected computer as anonymised images using the US applications such as ApliGate, developed by Canon for the secure

© Imperial College of Science, Technology and Medicine

sharing of images. Ultrasound images will be stored with the unique study identifier for each participant, the date of the scan and the initials of the person performing the examination.

For single cell RNA-seq experiments, we anticipate capturing ~3,000-5,000 cells per sample after quality control (QC) and filtering. Sequencing, batch correction, filtering, QC, doublet removal, ambient RNA correction and correction for multiple testing will be performed according to single-cell RNA-seq best practices using established pipelines and tools (e.g. https://github.com/DendrouLab; COMBAT Consortium 2022). Statistical and outcome measures will typically be assessed through ANOVA, Wilcoxon rank, and Fisher exact tests. Dimensionality reduction processes will be used to understand data structure and to identify clusters and cell types for statistical testing. Differential cluster abundance and differential gene expression using pseudobulk counts will be performed using relevant edgeR packages and applying a Benjamini-Hochberg multiple testing correction. Power calculations for single-cell RNA-seq data analysis are based on relevant available data (e.g., Turner et al. 2020), and for assessing differential abundance and gene expression, for instance, we estimate that n=10 individuals per group are required to have >80% power to detect 30% differences between longitudinal samples (pre and post vaccination), with a false discovery rate of 5%. Given that there is expected variation in sample yield after fine needle aspiration which may be anatomically determined, there is a 50% overage in each group to allow for this and for any participants lost to follow up bringing the total to n=30.

Single-cell repertoire analyses will be performed as previously described[23,24,25]. Briefly, T cell receptor (TCR) and B cell receptor (BCR) outputs from CellRanger will be further filtered and processed to remove homotypic doublets and low-quality droplets and annotated using IMGT. Single-cell TCR clonality measurements will include Shannon diversity calculation using the entropy R package, and mean clone size estimation by bootstrapped down-sampling. Single-cell BCR clonality measurements will include clonal expansion index calculation based on the Gini index of the number of total BCRs per clone, whilst the clonal diversification index is calculated as the Gini index of the number of unique BCRs per clone.

For bulk sequencing, standard pipelines will be used for data processing (COMBAT Consortium 2022). Principal component analysis will be used for initial analyses of the normalised and filtered data. Differential expression analysis of the normalised data will be performed using the limma R package for example, with pathway enrichment analysis being performed using the Reactome pathways via the XGR R package with Fisher’s exact test. Weighted gene correlation network analysis will be applied to identify modules of highly correlated genes. Bulk repertoire analyses will include filtering for base quality amongst other quality control checks, followed by TCR chain/Ig isotype frequency estimates and usage, CDR3 length characterisation, and somatic hypermutation and BCR class-switching analyses.

For flow cytometric analyses, data will be analysed using FlowJo, with appropriate control/validation with non-specific isotype controls and beads to ensure stable equipment calibration over time, as well as use of gating templates. For functional/serological analyses, assays will be performed in duplicate or triplicate with appropriate repeat experiments to ensure reproducibility of findings. Appropriate tests (e.g., t-tests, Wilcoxon rank-sum tests) for assessing inter-group differences will be performed using R, python or GraphPad Prism as required.

Data generated and all appropriate documentation will be stored for a minimum of 10 years after the completion of the study, including the follow-up period.

© Imperial College of Science, Technology and Medicine

13. REGULATORY ISSUES

ETHICS APPROVAL

The Study Coordination Centre will obtain approval from an NHS Research Ethics Committee (REC) and Health Research Authority (HRA). The study must also receive confirmation of capacity and capability from Imperial College Healthcare NHS Trust before accepting participants into the study or any research activity is carried out. The study will be conducted in accordance with the recommendations for physicians involved in research on human subjects adopted by the 18th World Medical Assembly, Helsinki 1964 and later revisions.

CONSENT

Consent to enter the study must be sought from each participant only after a full explanation has been given, an information leaflet offered, and time allowed for consideration. Signed participant consent should be obtained. The right of the participant to refuse to participate without giving reasons must be respected. After the participant has entered the study, the clinician remains free to give alternative treatment to that specified in the protocol or to withdraw participant from lymph node sample donation at any stage if he/she feels it is in the participant's best interest, but the reasons for doing so should be recorded. In these cases, the participants remain within the study for the purposes of follow-up and data analysis. All participants are free to withdraw at any time from the protocol treatment without giving reasons and without prejudicing further treatment.

CONFIDENTIALITY

Definition of terms

Pseudonymised data can be linked to an individual e.g., through a coded study key kept at the NHS study site.

Anonymised data cannot be linked directly back to an individual e.g., aggregated data for publication.

The Chief Investigator will preserve the confidentiality of participants taking part in the study and is registered under the Data Protection Act.

Individuals will receive a single non-transferable study code when attending screening for the study and keep the same code upon enrolment to the study. Data collected for the study will be pseudonymised by means of a coded study key kept at the NHS study site (Imperial College Healthcare NHS Trust. Pseudonymised data will be transferred from the study site to Imperial College London and University of Oxford for analysis without the study key. Anonymised data will be published.

© Imperial College of Science, Technology and Medicine

14. INDEMNITY

Imperial College London holds negligent harm and non-negligent harm insurance policies which apply to this study. Imperial College Healthcare NHS Trust holds standard NHS Hospital Indemnity and insurance cover with NHS Resolution for NHS Trusts in England, which apply to this study.

SPONSOR

Imperial College London will act as the main Sponsor for this study. Delegated responsibilities will be assigned to Imperial College Healthcare NHS Trust.

15. FUNDING

The Chan Zuckerberg Initiative is funding this study. Participants will be paid for each visit they complete, for their inconvenience and travel, at the end of their participation in the study, as follows:

- Screening (V1): £10
- Visits which include an FNA (V2, V4 and V6): £120
- Vaccination visit (V3): £80
- Follow-up visit V5: £60

16. STUDY MANAGEMENT

The day-to-day management of the study will be co-ordinated through NIHR Imperial CRF and the study manager Aime Palomeras.

LABORATORY DATA RECORDING AND MONITORING

Laboratory data management for the study will be co-ordinated by Calli Dendrou, Brian Marsden and Teresa Lambe at the University of Oxford. Data will be stored with linked anonymised (pseudonymised) codes, and all files and databases will be password protected and held on password protected computers. Pseudanonymised identifiers will be kept on a secured NHS workstation at NIHR Imperial CRF before transfer of the minimally required demographic metadata (e.g. sex and age) to study scientists in Oxford.

Raw and analysed sequencing, genetic, flow cytometric and serological data will be collated and stored on a restricted access site on a University of Oxford computational cluster (BMRC) and will be managed and curated according to established protocols. The storage system of the computational cluster is resilient to disk failure and data are automatically backed up to linked off-site servers. Data files will be clearly labelled and associated (cleaned and anonymised) metadata will be stored in tab-delimited text files so as to document the provenance of the data. Where automated analyses are performed, these will be version controlled and the commands used will be recorded in log files. Data arising from smaller-scale laboratory analyses will be recorded in hard copy or electronic laboratory notebooks according to standard University practices. Data (and analysis scripts) will be maintained in this way for ten years after study completion.

© Imperial College of Science, Technology and Medicine

PROJECT MANAGEMENT AND GOVERNANCE

Imperial College London and University of Oxford are the principal partners for delivery of the study in the UK. These two institutions will partner with Uganda Virus Research Institute (UVRI), who will be responsible for international capacity building to undertake future single-cell and lymphatic tissue focussed vaccine research under the umbrella of the LEGACY Network.

17. PATIENT AND PUBLIC INVOLVEMENT

Patient and Public Involvement and Engagement (PPIE) in research is research being carried out 'with' or 'by' members of the public rather than 'to', 'about' or 'for' them. The term "patient and public" includes patients, participants, carers and people who use health and social care services as well as people from specific communities and from organisations that represent people who use services.

The protocol has been reviewed and approved by the LEGACY01 PPIE committee.

18. PUBLICATION POLICY

The preparation of a manuscript for publication in a peer-reviewed professional journal or an abstract for presentation, oral or written, to a learned society or symposium will be discussed on the study calls and with the PPIE Advisory Group. Details of dissemination can be found in the study specific communication plan.

Authorship will reflect work done by the investigators and other personnel involved in the analysis and interpretation of the data, in accordance with generally recognised principles of scientific collaboration.

19. DATA AND SAMPLE SHARING

The protocol will be published and made open access during the study.

During the study, data will be shared through controlled access using the following principles:

No data should be released that would compromise an ongoing trial or study.

There must be a strong scientific or other legitimate rationale for the data to be used for the requested purpose.

Investigators have a period of exclusivity in which to pursue their aims with the data before data are made available to other researchers.

Adequate resources must be available in order to comply in a timely manner or at all, and the scientific aims of the study must justify the use of such resources.

Data exchange complies with Information Governance and Data Security Policies in all the relevant countries.

Researchers wishing to access study data should contact the chief investigator in the first instance.

© Imperial College of Science, Technology and Medicine

New algorithms or methods of value to the broader community will be shared online via the GitHub and/or protocols.io websites.

At the end of the study, single-cell data will be archived and made open access as part of the Human Cell Atlas data portal (https://data.humancellatlas.org/), and other data (e.g., imaging, bulk RNA sequencing, genotyping, flow cytometric, and serological data) will be made open access through deposition in the European Genome-phenome Archive (EGA; https//ega-archive.org), Cytobank (https://mrc.cytobank.org/cytobank/) and Zenodo (https://zenodo.org), for example. Meta-data deposited along with the sequencing data will be MINSEQE-compliant.

20. PROTOCOL AMENDMENTS

The protocol v2.0 will be the first version approved for use.

Protocol v3.1 is the first substantial amendment and includes addition of an optional visit 6, addition of a participant feedback survey, a change to the maximum body max index allowable in the study, an update to the seasonal influenza vaccine for use for the season 2023-2024, updated referencing, addresses and study registration, and correction of minor errors in typing.

© Imperial College of Science, Technology and Medicine

21. REFERENCES

1. Huang C, Wang Y, Li X, et al. (2020) Clinical Features of Patients Infected With 2019 Novel Coronavirus in Wuhan, China. Lancet 395:497-506.
2. Petersen LR, Jamieson DJ, Powers AM, Honein MA (2016) Zika virus. N Engl J Med 374:1552-1563.
3. WHO Ebola Response Team (2014) Ebola virus disease in West Africa—the first 9 months of the epidemic and forward projections. N Engl J Med 371:1481-1495.
4. WHO Disease Outbreak News, https://www.who.int/emergencies/disease-outbreak-news
5. Ball P (2021) The lightning-fast quest for COVID vaccines – and what it means for other diseases. Nature 589:16-18.
6. Christy C, Pichichero ME, Reed GF, et al. (1995) Effect of gender, race, and parental education on immunogenicity and reported reactogenicity of acellular and whole-cell pertussis vaccines. Pediatrics 96:584-587.
7. Kurupati R, Kossenkov A, Haut L, et al. (2016) Race-related differences in antibody responses to the inactivated influenza vaccine are linked to distinct pre-vaccination gene expression profiles in blood. Oncotarget 7:62898-628911.
8. Haralambieva IH, Salk HM, Lambert ND, et al. (2014) Associations between race, sex and immune response variations to rubella vaccination in two independent cohorts. Vaccine 32:1946-1953.
9. Sharma S, Hagbom M, Svensson L, Nordgren J (2020) The impact of human genetic polymorphisms on rotavirus susceptibility, epidemiology, and vaccine take. Viruses 12:324.
10. D'Souza RS, Wolfe I (2021) COVID-19 vaccines in high-risk ethnic groups. Lancet 397:1348.
11. Peng K, Safonova Y, Shugay M (2021) Diversity in immunogenomics: the value and the challenge. Nat Methods doi: 10.1038/s41592-021-01169-5.
12. Razai MS, Osama T, McKechnie DGJ, Majeed A (2021) COVID-19 vaccine hesitancy among ethnic minority groups. BMJ 372:n513.
13. Liang F, Lindgren G, Sandgren KJ et al. (2017) Vaccine priming is restricted to draining lymph nodes and controlled by adjuvant-mediated antigen uptake. Sci Transl Med 9:eaal2094.
14. Cirelli KM, Carnathan DG, Nogal B et al. (2019) Slow delivery immunization enhances HIV neutralizing antibody and germinal center responses via modulation of immunodominance. Cell 177:1153-1171.
15. Havenar-Daughton C, Carnathan DG, Torrents de la Peña A et al. (2016) Direct probing of germinal center responses reveals immunological features and bottlenecks for neutralizing antibody responses to HIV Env trimer. Cell Rep 17:2195-2209.
16. Havenar-Daughton C, Newton IG, Zare SY et al. (2020) Normal human lymph node T follicular helper cells and germinal center B cells accessed via fine needle aspirations. J. Immunol. Methods 479:112746.
17. Turner JS, Zhou JQ, Han J (2020) Human germinal centres engage memory and naïve B cells after influenza vaccination. Nature 586:127-132.
18. https://www.humancellatlas.org/
19. https://www.ema.europa.eu/en/documents/product-information/fluad-tetra-epar-product-information_en.pdf

© Imperial College of Science, Technology and Medicine

20. https://www.fda.gov/media/135686/download
21. Fluad, Suspension for injection in pre-filled syringe - Summary of Product Characteristics (SmPC) - (emc) (medicines.org.uk)
22. Day S, Kaur C, Cheeseman HM, et al. (2022) Comparison of blood and lymph node cells after intramuscular injection with HIV envelope immunogens. Front. Immunol. 13:991509.
23. Corridoni D, Antanaviciute A, Gupta T, et al. (2020) Single-cell atlas of colonic CD8+ T cells in ulcerative colitis. Nat Med 26: 1480-1490.
24. Huang B, Chen Z, Geng L, et al. (2019) Mucosal profiling of pediatric-onset colitis and IBD reveals common pathogenics and therapeutic pathways. Cell 179: 1160-1176.
25. COvid-19 Multi-omics Blood ATlas (COMBAT) Consortium. (2022) A blood atlas of COVID-19 defines hallmarks of disease severity and specificity. Cell 185: 916-938.

© Imperial College of Science, Technology and Medicine

22. APPENDIX 1. INFLUENZA VACCINE USE IN THE UK

Table S1 All influenza vaccines marketed in the UK for the 2023 to 2024 season (as of 17 Apr 2023)

**Supplier**	**Name of product**	**Vaccine type**	**Age indications**	**Ovalbumin content micrograms per dose**	**Contact details**
AstraZeneca UK Ltd	Fluenz® Tetra	Quadrivalent LAIV (live attenuated influenza vaccine) supplied as nasal spray suspension	From 24 months to less than 18 years of age	Less than 0.024 micrograms per 0.2 ml dose	0845 139 0000
Sanofi	Quadrivalent influenza vaccine	QIVe (standard egg-grown quadrivalent influenza vaccine), split virion, inactivated	From 6 months	Equal to or less than 0.05 micrograms per 0.5 ml dose	0800 854 430
Viatris	Influvac® sub-unit Tetra ▼	QIVe (standard egg-grown quadrivalent influenza vaccine), surface antigen, inactivated	From 6 months	Equal to or less than 0.1 micrograms per 0.5 ml dose	0800 358 7468
Seqirus UK Ltd	Cell-based quadrivalent influenza vaccine Seqirus ▼	QIVc (cell-grown quadrivalent influenza vaccine), surface antigen, inactivated	From 2 years	Egg-free	08457 451 500
Sanofi	Supemtek ▼	QIVr (quadrivalent influenza vaccine (recombinant, prepared in cell culture))	From 18 years	Egg-free	0800 854 430
**Seqirus UK Ltd**	**Adjuvanted Quadrivalent Influenza Vaccine Seqirus ▼**	**aQIV (adjuvanted egg-grown quadrivalent influenza vaccine) surface antigen, inactivated, adjuvanted with MF59C.1**	**From 65 years**	**Equal to or less than 1 micrograms per 0.5 ml dose**	**08457 451 500**

The vaccine to be used in LEGACY01 study is in bold text.

Source: https://www.gov.uk/government/publications/influenza-vaccines-marketed-in-the-uk/all-influenza-vaccines-marketed-in-the-uk-for-the-2022-to-2023-season

© Imperial College of Science, Technology and Medicine

Table S2 All influenza vaccines marketed in the UK for the 2022 to 2023 season (as of 14 Apr 2022)

**Supplier**	**Name of product**	**Vaccine type**	**Age indications**	**Ovalbumin content micrograms per dose**	**Contact details**
AstraZeneca UK Ltd	Fluenz® Tetra	Quadrivalent LAIV (live attenuated influenza vaccine) supplied as nasal spray suspension	From 24 months to less than 18 years of age	Less than 0.024 micrograms per 0.2 ml dose	0845 139 0000
MASTA	Quadrivalent influenza vaccine	QIVe (standard egg-grown quadrivalent influenza vaccine), split virion, inactivated	From 6 months	Equal to or less than 0.05 micrograms per 0.5 ml dose	0113 238 7552
MASTA	Quadrivalent Influvac® sub-unit Tetra ▼	QIVe (standard egg-grown quadrivalent influenza vaccine), surface antigen, inactivated	From 6 months	Equal to or less than 0.1 micrograms per 0.5 ml dose	0113 238 7552
Sanofi Pasteur	Quadrivalent influenza vaccine	QIVe (standard egg-grown quadrivalent influenza vaccine), split virion, inactivated	From 6 months	Equal to or less than 0.05 micrograms per 0.5 ml dose	0800 854 430
Viatris (formerly Mylan)	Quadrivalent Influvac® sub-unit Tetra ▼	QIVe (standard egg-grown quadrivalent influenza vaccine), surface antigen, inactivated	From 6 months	Equal to or less than 0.1 micrograms per 0.5 ml dose	0800 358 7468
Seqirus UK Ltd	Cell-based quadrivalent influenza vaccine Seqirus ▼	QIVc (cell-grown quadrivalent influenza vaccine), surface antigen, inactivated	From 2 years	Egg-free	08457 451 500
Sanofi Pasteur	Supemtek ▼	QIVr (quadrivalent influenza vaccine (recombinant, prepared in cell culture))	From 18 years	Egg-free	0800 854 430
**Seqirus UK Ltd**	**Adjuvanted Quadrivalent Influenza Vaccine Seqirus ▼**	**aQIV (adjuvanted egg-grown quadrivalent influenza vaccine) surface antigen, inactivated, adjuvanted with MF59C.1**	**From 65 years**	**Equal to or less than 1 micrograms per 0.5 ml dose**	**08457 451 500**

The vaccine to be used in LEGACY01 study is in bold text.
Source: https://www.gov.uk/government/publications/influenza-vaccines-marketed-in-the-uk/all-influenza-vaccines-marketed-in-the-uk-for-the-2022-to-2023-season

© Imperial College of Science, Technology and Medicine

Table S3 All influenza vaccines marketed in the UK for the 2021 to 2022 season (as of 23 Jun 2021)

Supplier	Name of product	Vaccine type	Age indications	Ovalbumin content micrograms/dose	Contact details
AstraZeneca UK Ltd	Fluenz® Tetra	Quadrivalent LAIV (live attenuated influenza vaccine) supplied as nasal spray suspension	From 24 months to less than 18 years of age	Less than 0.024 micrograms per 0.2 ml dose	0845 139 0000
MASTA	Quadrivalent Influenza vaccine	QIVe (standard egg-grown quadrivalent influenza vaccine), split virion, inactivated	From 6 months	Equal to or less than 0.05 micrograms per 0.5 ml dose	0113 238 7552
Sanofi Pasteur Vaccines	Quadrivalent Influenza vaccine	QIVe (standard egg-grown quadrivalent Influenza vaccine), split virion, inactivated	From 6 months	Equal to or less than 0.05 micrograms per 0.5 ml dose	0800 854 430
Viatris (formerly Mylan)	Quadrivalent Influvac® sub-unit Tetra ▼	QIVe (standard egg-grown quadrivalent Influenza vaccine), surface antigen, inactivated	From 6 months	Equal to or less than 0.1 micrograms per 0.5 ml dose	0800 358 7468
Seqirus UK Ltd	Flucelvax® Tetra ▼	QIVc (cell-grown quadrivalent Influenza vaccine), surface antigen, inactivated	From 2 years	Egg-free	08457 451 500
Sanofi Pasteur Vaccines	Supemtek ▼	QIVr (quadrivalent Influenza vaccine (recombinant, prepared in cell culture))	From 18 years	Egg-free	0800 854 430
Seqirus UK Ltd	Fluad Tetra ▼	aQIV (egg-grown quadrivalent Influenza vaccine), surface antigen, inactivated, adjuvanted with MF59C.1	From 65 years	Equal to or less than 1 micrograms per 0.5 ml dose	08457 451 500

Source: https://www.gov.uk/government/publications/influenza-vaccine-ovalbumin-content/influenza-vaccines-2020-to-2021-flu-season

COMPOSITION

One 0.5 mL dose of aQIV contains 15 micrograms of haemagglutinin from two A and two B strains of influenza propagated in hens' eggs and adjuvanted with MF59C.1 which contains per 0.5mL dose, squalene (9.75 mg), polysorbate 80 (1.175 mg), sorbitan trioleate (1.175 mg), sodium citrate (0.66 mg) and citric acid (0.04 mg).

By comparison, one 0.5 mL dose of Supemtek contains 45 micrograms of influenza virus haemagglutinin from two A strains and two B strains produced by recombinant DNA technology using a baculovirus expression system in an insect cell line derived from Spodoptera frugiperda.

© Imperial College of Science, Technology and Medicine

Table S3 Comparison of Supemtek and aQIV: safety data

	**Very common**<br><br>**(≥1/10)**	**Common**<br><br>**(≥1/100 to <1/10)**	**Uncommon**<br><br>**(≥1/1,000 to <1/100)**	**Rare**
aQIV: Adverse reactions reported following vaccination in elderly subjects 65 years and older in clinical trials	Headache, injection site pain, fatigue	Nausea, diarrhoea, myalgia, arthralgia, ecchymosis, chills, erythema, induration, ILI	Vomiting, fever ≥38C	
Supemtek Adverse reactions reported following vaccination in adults 18 years and older during clinical trials and post-marketing surveillance	Headache, fatigue, myalgia, arthralgia, local tenderness,<br><br>local pain	Nausea, firmness / swelling, redness, fever, shivering / chills,	Cough,<br><br>oropharyngeal pain, diarrhoea, pruritus, dermatitis, rash flu-like symptoms, injection site pruritus,	Dizziness, urticaria

aQIV: no post marketing data are yet available. Fluad trivalent formulation has post marketing reports of thrombocytopaenia, lymphadenopathy, extensive limb swelling, allergy/anaphylaxis, angioedema, muscular weakness, Encephalomyelitis, Guillain-Barré syndrome, convulsions, neuritis, neuralgia, paraesthesia, generalised skin reactions including erythema multiforme, urticaria, pruritus or non-specific rash, and vasculitis with transient renal involvement.

Supemtek: Hypersensitivity including anaphylaxis has been reported with an unknown frequency. Guillain-Barre syndrome has been reported with an unknown frequency and a causal relationship has not been established.

© Imperial College of Science, Technology and Medicine

Table S4 Comparison of Supemtek and aQIV: immunogenicity data in older adults*

Lineage	A	A	B	B
	A/H1N1	A/H3N2	B/Yamagata	B/Victoria
aQIV: 65 years and older GMT	65.0 (57.8; 73.1)	294.9 (261.9; 332.1)	24.7 (22.7; 26.8)	30.8 (28.3; 33.5)
aQIV: 65 years and older seroconversion rate	35.2 (32.0; 38.5)	39.3 (36.1; 42.7)	16.4 (14.0; 19.0)	13.4 (11.2; 15.9)
Supemtek adults > 50 years GMT	A/California/7/2009 (H1N1)	A/Texas/50/2012 (H3N2)	B/Massachusetts/02/ 2012 (Yamagata lineage)	B/Brisbane/60/2008 (Victoria lineage)
	190 (164;221)	522 (462;589)	55 (48;64)	29 (26;33)
Supemtek adults > 50 years seroconversion rate	44.9 (39.3; 50.6)	54.5 (48.8; 60.1)	38.9 (33.4; 44.5)	21.0 (16.6; 25.9)

*immunogenicity data for younger adults are not on the SmPC for aQIV

Supemtek solution for injection in pre-filled syringe - Summary of Product Characteristics (SmPC) - (emc) (medicines.org.uk)

Adjuvanted Quadrivalent Influenza Vaccine (Surface Antigen, Inactivated) Seqirus suspension for injection in pre-filled syringe Influenza vaccine, Adjuvanted with MF59C.1 - Summary of Product Characteristics (SmPC) - (emc) (medicines.org.uk)

© Imperial College of Science, Technology and Medicine

23. APPENDIX 2. FINE NEEDLE ASPIRATION OF THE LYMPH NODE

A medical practitioner will carry out the FNA using clinical facilities at Imperial College Healthcare NHS Trust, London, UK. Eligibility to undergo the procedure will be confirmed, paying attention to

Blood thinning medication likely to induce bruising taken prior to aspiration

Signs of local infection

Pain or swelling at any sites of potential lymph node sampling

Allergy to local anaesthetic

Any other medical reason, which the PI deems significant to warrant exclusion from the FNA

Participants will have a set of observations performed including temperature, blood pressure and pulse rate.

The FNA will be conducted using standard aseptic technique under ultrasound guidance. During the procedure, the ipsilateral and contralateral lymph nodes in the axilla will be located by physical examination of the full lymphatic system, and then under US guidance. A sterile needle and syringe will be used to aspirate material from lymph nodes on each side using 3-5 passes. Where necessary local anaesthesia will be employed to numb the area prior to sampling, using a standard local anaesthetic e.g., 1% lidocaine.

At each visit for FNA sampling a paired peripheral blood sample will be taken using standard non touch aseptic phlebotomy technique.

Lymph node samples will be placed into pre-prepared and labelled specimen pots and placed with the blood tubes in an appropriate transportation container. They will be transferred to the receiving laboratory where they will be processed upon receipt. The equipment necessary will all be made available on the day, including an US machine, and equipment for FNA (disinfectant, local anaesthetic, needles, 5ml syringes, air-tight specimen tubes prepared with R10 transport medium).

Participants will be observed for a minimum of 30 minutes after the procedure. There will be an AE check and FNA site inspection at least 30 minutes post-FNA.

EXPECTED ADVERSE EVENTS AND GRADING

Expected adverse events following lymph node aspiration include sample site pain or tenderness. Haematoma is a rare risk, and minimal bleeding may occur after the aspiration but should resolve spontaneously, and participants at increased risk due to blood-thinning medication will be excluded. Bruising may occur but is expected to fade after 2 weeks. Participants will be provided with information regarding expected adverse events in a participant information leaflet and adverse events will be monitored and reported as per standard AE reporting for the LEGACY01 study.

© Imperial College of Science, Technology and Medicine

24. APPENDIX 3. POST MARKETING SURVEILLANCE FOR FLUAD

Adverse reactions reported in post marketing surveillance of the aTIV, FLUAD include thrombocytopenia including severe thrombocytopaenia in very rare cases, lymphadenopathy, asthenia, Influenza-Like Illness (ILI), swelling and redness of injected limb, allergic reactions including, rarely anaphylactic shock, anaphylaxis and angioedema, pain in the extremity, muscular weakness, encephalomyelitis, Guillain-Barré Syndrome, convulsions, neuritis, neuralgia, paraesthesia, syncope, presyncope, generalised skin reactions including erythema multiforme, urticaria, pruritus or non-specific rash, vasculitis which possibly associated with transient renal involvement.

https://www.medicines.org.uk/emc/product/9223/smpc

© Imperial College of Science, Technology and Medicine
